# Modelling post-chemotherapy stem cell dynamics in the bone marrow niche of AML patients

**DOI:** 10.1038/s41598-024-75429-7

**Published:** 2024-10-23

**Authors:** Chenxu Zhu, Thomas Stiehl

**Affiliations:** 1 Institute for Computational Biomedicine-Disease Modeling, RWTH Aachen University, Aachen, Germany; 2 Department of Science and Environment, Roskilde University, Roskilde, Denmark; 3 Centre for Mathematical Modeling-Human Health and Disease, Roskilde University, Roskilde, Denmark

**Keywords:** Acute myeloid leukemia (AML), 7+3 chemotherapy, Stem cell niche, Cancer stem cell, Computational model, Mathematical model, Cancer models, Acute myeloid leukaemia, Applied mathematics

## Abstract

Acute myeloid leukemia (AML) is a stem cell-driven malignancy of the blood forming (hematopoietic) system. Despite of high dose chemotherapy with toxic side effects, many patients eventually relapse. The “7+3 regimen”, which consists of 7 days of cytarabine in combination with daunorubicin during the first 3 days, is a widely used therapy protocol. Since peripheral blood cells are easily accessible to longitudinal sampling, significant research efforts have been undertaken to characterize and reduce adverse effects on circulating blood cells. However, much less is known about the impact of the 7+3 regimen on human hematopoietic stem cells and their physiological micro-environments, the so-called stem cell niches. One reason for this is the technical inability to observe human stem cells in vivo and the discomfort related to bone marrow biopsies. To better understand the treatment effects on human stem cells, we consider a mechanistic mathematical model of the stem cell niche before, during and after chemotherapy. The model accounts for different maturation stages of leukemic and hematopoietic cells and considers key processes such as cell proliferation, self-renewal, differentiation and therapy-induced cell death. In the model, hematopoietic (HSCs) and leukemic stem cells (LSCs) compete for a joint niche and respond to both systemic and niche-derived signals. We relate the model to clinical trial data from literature which longitudinally quantifies the counts of hematopoietic stem like (CD34+CD38-ALDH+) cells at diagnosis and after therapy. The proposed model can capture the clinically observed interindividual heterogeneity and reproduce the non-monotonous dynamics of the hematopoietic stem like cells observed in relapsing patients. Our model allows to simulate different scenarios proposed in literature such as therapy-related impairment of the stem cell niche or niche-mediated resistance. Model simulations suggest that during the post-therapy phase a more than 10-fold increase of hematopoietic stem-like cell proliferation rates is required to recapitulate the measured cell dynamics in patients achieving complete remission. We fit the model to data of 7 individual patients and simulate variations of the treatment protocol. These simulations are in line with the clinical finding that G-CSF priming can improve the treatment outcome. Furthermore, our model suggests that a decline of HSC counts during remission might serve as an indication for salvage therapy in patients lacking MRD (minimal residual disease) markers.

## Introduction

Acute myeloid leukemia (AML) is an aggressive blood cancer characterized by the expansion of aberrant myeloid blasts and an impairment of healthy blood cell formation^1^. Involvement of the stem cell niche triggers the loss of healthy hematopoietic stem cells (HSCs) and a subsequent reduction of circulating functional blood cells^2^.

The “7+3” chemotherapy regimen is often considered as the standard intervention for remission induction in adulthood AML^3^. It is comprised of cytarabine (AraC) and daunorubicin (DNR). AraC is applied during seven days and on the first three days in combination with daunorubicin (DNR)^4^. As an antimetabolic agent, AraC blocks the function of DNA polymerases^5^ and kills mitotic cells. Thus, it acts preferentially on leukemic cells, which are assumed to have a high proliferation rate. Combined with DNR, which inhibits leukemic cells’ expansion through both anti-mitotic and cytotoxic modes of action^6^, a further reduction of the malignant cell burden can be achieved. Due to the limited accessibility of human bone marrow, the precise dynamics of HSCs and leukemic stem cells (LSCs) after chemotherapy are not well understood.

Combining mathematical models with clinical and experimental data can provide insights into processes, which cannot be observed directly. Mathematical modeling has substantially contributed to the understanding of the hematopoietic system and its diseases. Pioneering works on the mechanistic origin of oscillating blood cell counts and the growth dynamics of leukemic cells date back to the 1970s^7–10^. Since then, various aspects of the hematopoietic system have been studied with the help of *in silico* approaches. For example, computational models have been used to quantify human steady state HSC numbers and replication rates^11,12^. Age- and stress- related changes of blood cell production have been investigated in^13–15^. Feedback regulations contributing to the stabilization of the homeostatic state have been considered in^16–19^. In the context of AML, mathematical modelling has been utilized to understand important disease mechanisms and to simulate the treatment outcome. For example, the works^20,21^ aim to predict the dynamics of AML relapse and patient prognosis and the models in^22–24^ focus on blood cell formation after AML chemotherapy. Reference^25^ investigates links between the proliferative advantage of AML cells, chemosensitivity and time to relapse. The impact of the bone marrow microenvironment (niche) on stem cell regulation has also been studied with the help of mathematical models^25–31^. Models of the therapy of chronic lymphoid leukemia, chronic myeloid leukemia and phiadelphia negative myeloproliferative neoplasms have been developed in references^32–35^.

The focus of this work is to understand HSC and progenitor dynamics in AML patients during and after chemotherapy. From long-term survivors after bone marrow transplantation, it is known that patients with reduced HSC counts can exhibit normal marrow cellularity and physiological peripheral blood cell counts^36^. Therefore, routine clinical samples, even if acquired longitudinally, might provide only limited insights in the dynamics of the stem cells and early progenitors.

The main novelty of this work is to combine a mechanistic computational model of the stem cell niche with quantitative longitudinal data of stem like (CD34+CD38-ALDH+) hematopoietic cells. The CD34+CD38-ALDH+ subset is mainly composed of LT-HSC (Long-term HSC), ST-HSC (Short-term HSC) and multipotent progenitors (MPPs) and in so-called ALDH-rare AML (a disease subtype comprising approximately 75% of all AML cases) devoid of leukemic cells^28,37,38^. As this cell fraction is highly enriched for HSCs, we utilize it to quantify HSC dynamics in our mathematical model. Since this cell fraction is not routinely measured, the amount of available data is small. Nevertheless, it can provide important insights into the dynamics of the most primitive hematopoietic cells in human AML. We use the data from a clinical study by Wang et al, which investigates the time course of stem like hematopoietic cells and AML blasts^28^.

In our study, we extend a model of the human stem cell niche^26,39^ to account for the effects of 7+3 chemotherapy. The model describes the time evolution of multiple healthy and leukemic cell types which are subjected to systemic feedbacks and regulatory cues from the stem cell niche. We propose an additional feedback signal which is able to explain the longitudinal HSC dynamics and the observed inter-individual heterogeneity in response to chemotherapy. We aim to provide insights into the following questions: (1) How do the dynamics of stem-like cells change in response to therapy? (2) What are potential differences between relapsing and non-relapsing patients? (3) How does G-CSF priming affect the therapy outcome? (4) What are suitable indications of salvage therapy in patients lacking an MRD (minimal residual disease) marker? (5) How could a therapy-related injury of the stem cell niche impact therapy response?

## Methods

### Model of chemotherapy

**Figure 1:**
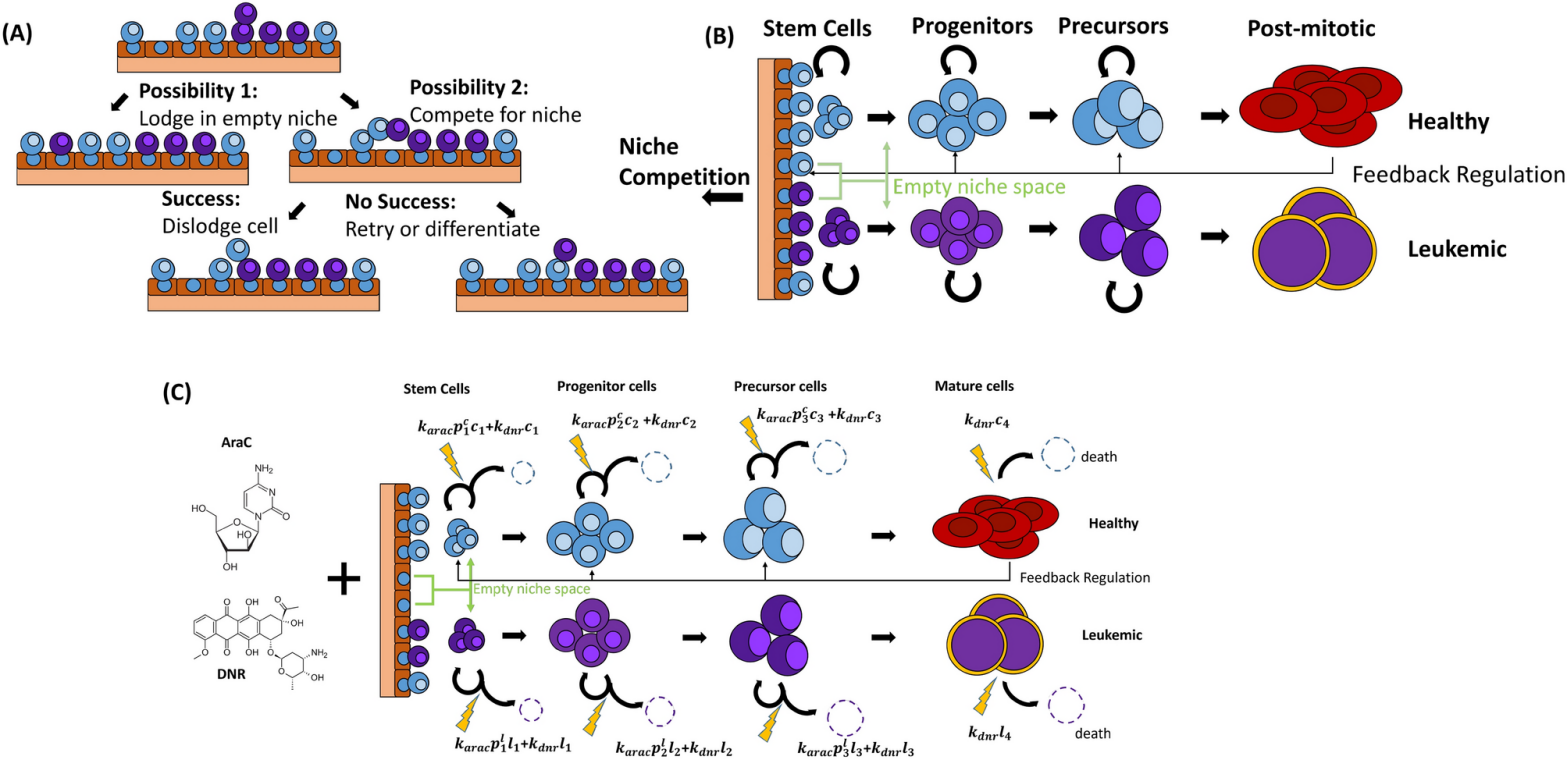
Model of acute myeloid leukemia (AML) and “7+3” chemotherapy. (**A**) illustrates the stem cells competition inside bone marrow niche. (**B**) summarizes the considered cell types of the healthy and malignant lineage together with the two nonlinear feedbacks. The black line denotes the systemic feedback signal which depends on the amount of circulating cells and the green line corresponds to the micro-environmental regulation which depends on the frequency of empty niche spaces. (**C**) illustrates the model of “7+3” chemotherapy with cytarabine (AraC) and daunorubicin (DNR). Based on the drug mechanism, AraC acts on stem, progenitor and precursor cells, DNR acts on all four cell states.

As point of departure we use the AML model from^26,39^ and extend it to account for 7+3 chemotherapy. Main features of the model are summarized in Fig. 1A, B and Supplemental Fig. S1. The complete derivation is provided in Supplement S1. This model accounts for one hematopoietic cell lineage consisting of stem, progenitor, percursor and mature cells and one leukemic cell lineage, consisting of leukemic stem, progenitor, precursor cells and post-mitotic blasts. HSCs and LSCs compete for spaces in a joint stem cell niche of fixed capacity *K*, which they require to maintain stemness. Upon division, one stem cell gives rise to two offspring, one of which occupies the niche space of the parent, the other attempts to enter a randomly chosen niche space. If this niche space is not empty, the cell occupying it can be dislodged. The probability by which LSCs dislodge HSCs from the niche is referred to as *HSC dislodgement probability *$q_L$. If the dislodgement is successful, the daughter cell maintains its stemness and the dislodged cell differentiates. If the daughter cell fails to enter the niche within a predefined number of attempts, it differentiates. The higher the number of empty niche spaces, the higher the probability that offspring of dividing stem cells remain stem cells. In this sense, the stem cell niche regulates stem cell self-renewal.

Differentiated stem cells enter the progenitor state. Unlike stem cells, progenitors cannot self-renew indefinitely and differentiate into precursors after a finite number of divisions. Analogously, leukemic and hematopoietic precursors differentiate into post mitotic cells (mature cells or post-mitotic blasts, respectively) after a finite number of divisions. Post-mitotic cells are assumed to be cleared at a constant rate. This setup has been shown to be able to recapitulate AML dynamics and to provide clinically meaningful insights into disease progression^26,39^.

As in previous works by us and others, we assume that the drug-induced cell death is proportional to the drug dose and the number of target cells^40,41^. Our model is based on the following biologically motivated assumptions:

#### Assumption 1

The rate of AraC - induced cell death at time *t* is proportional to the number of cycling cells at time *t*. For LSCs it is modeled by $k_{arac}p^l_{1}(t)l_{1}(t)$, where $p_{1}^l(t)$ is the LSC proliferation rate and $l_1(t)$ the size of the LSC population at time *t*. The dimensionless factor $k_{arac}$, referred to as the drug effect parameter, quantifies the treatment effect. This choice is motivated by the fact that AraC acts mainly on the cells in the DNA synthesis phase^42^. Analogous terms are used for all other mitotic healthy and leukemic cell types, Fig. 1C. We, furthermore, assume that $k_{arac}$ is the same for all mitotic cells. Allowing the drug effect parameter to differ between healthy and malignant cells, does not improve the model fit.

#### Assumption 2

AraC has no impact on mature blood cells and post-mitotic leukemic blasts.

#### Assumption 3

There is evidence that DNR exerts cell cycle-dependent and cell cycle-independent toxicities^6^. Therefore, it is challenging to quantify the DNR-induced death rates for the different cell types^43^. To keep the number of model parameters as small as possible and since we already account for cell cycle-dependent toxicity in the context of AraC, we focus on the cell cycle-independent cytotoxicity of DNR. We model the DNR effect by a constant drug effect parameter $k_{dnr}$ which is assumed to be the same for all mitotic and post-mitotic cell types. For LSCs the DNR-induced death is $k_{dnr}l_1(t)$, for other cell types it is analogous. If the abundance of a cell population is below one cell per kg of body weight, we consider the respective cell population as extinct, as in^41,44^.

### Regulation of stem cell dynamics by systemic and micro-environmental signals

We assume that stem cell kinetics are regulated by local and systemic signals^45^. In our model, stem cell self-renewal is regulated by the stem cell niche: the more empty niche spaces exist, the higher the probability that offspring of dividing stem cells home to the niche and maintain their stemness. This is in line with observations showing that HSC self-renewal is impaired in *in vitro* assays lacking an appropriate micro-environment^46^. For HSC proliferation we consider regulation by micro-environmental and systemic signals. As a prototypic example for systemic signals we take G-CSF which reflects the mature neutrophil counts^47^ and acts on HSCs^48^ and progenitors/precursors^49^.

By $s_1(t)$ we denote the strength of the systemic proliferative signal at time *t*:

1 $$\begin{aligned} {s}_{1}(t) = \frac{1}{1+k_p c_{4}(t)} \end{aligned}$$

It depends on the mature cell count $c_4(t)$, $k_p$ is a positive constant. The signal assumes the maximal value one in absence of mature cells. Hill functions are widely used to model feedback signals in the hematopoietic system^50–54^ and can be rigorously derived based on time scale separation^55^.

In addition, we consider a signal $s_2(t)$ which emerges from the stem cell niche. This signal is modeled by a Hill function which increases for increasing numbers of empty niche spaces. The abundance of empty niche spaces at time *t* is given by $K-c_1(t)-l_1(t)$, where *K* represents the total amount of niche spaces per kg of body weight and $c_1(t)$ and $l_1(t)$ denote the number of hematopoietic and leukemic stem cells per kg of body weight, respectively:

2 $$\begin{aligned} {s}_{2}(t) = \left( \frac{K-c_{1}(t)-l_{1}(t)}{1+K-c_{1}(t)-l_{1}(t)}\right) /\left( \frac{K}{1+K}\right) . \end{aligned}$$

When the niche is empty ($c_{1}(t) = l_{1}(t) =0$), the signal assumes its maximum value 1, when the niche is completely filled ($c_{1}(t) + l_{1}(t) = K$) it assumes the minimum 0. Note that it holds $0\le c_{1}(t) + l_{1}(t) \le K$.

How cells integrate different signals into a response is not well understood^56^. We assume that HSCs integrate both signals $s_1(t)$ and $s_2(t)$ into a proliferative response ${\tilde{s}}_{hsc}(t)$:

3 $$\begin{aligned} \widetilde{s}_{hsc}(t)~&= \left\{ \begin{array}{rcl} {s}_{max}\cdot {s}_{2}(t) & & {{s}_{1}(t)~ < {s}_{2}(t)~} \\ {s}_{1}(t) & & {{s}_{1}(t)~ \ge {s}_{2}(t)~} \end{array}\right. \end{aligned}$$

where $s_{max}$ is a positive constant corresponding to the maximal possible increase of the proliferation rate in response to the micro-environmental signal. The HSC proliferation rate at time *t* is $p_1^c(t)={\tilde{p}}_1^c\cdot \tilde{s}_{hsc}(t)$ and ${\tilde{p}}_1^c$ corresponds to the proliferation rate for $s_1(t)=1$.

The motivation of this expression is as follows: We observe that in the post-chemotherapy period, HSC expansion is highly increased compared to the equilibrium proliferation rate of one to two divisions per year^11^. We fitted an exponential growth curve through the two initial measurements of CD34+CD38-ALDH+ cells of patients 3, 7, and 10 from^28^. These measurements account for the expansion of stem like cells in the immediate aftermath of chemotherapy. The obtained growth rates are 0.0655/*day*, corresponding to one division each 10.6 days (Patient 3), 0.1731/*day*, corresponding to one division each 4 days (Patient 7) and 0.0329/*day*, corresponding to one division each 21.1 days (Patient 10). Division frequencies were calculated as $\log (2)$ divided by the growth rate. It is well established that specific stress mechanisms such as inflammatory signals can increase HSC proliferation after chemotherapy^57^.

There exist contradicting results on the question whether peripheral blood cell loss induces HSC cycling^58,59^. This might suggest that effects, if they exist, are rather mild. Therefore, we assume that the more than 10-fold increase of proliferation rate observed after chemotherapy might be mediated by the niche, i.e., the signal $s_2$. Opposed to this, the systemic signal $s_1$ is responsible for the steady state HSC proliferation and allows a mild demand-related change of the proliferation rate.

It is known that after bone marrow transplantation, the fraction of stem like cells (CD34+CD90+) in S/G2/M is increased approximately 4 fold^60^. We assume that after transplantation (when a large proportion of the niche is probably refilled by donor cells), the effect of the niche-derived signal $s_2$ can be neglected. Therefore, we attribute the observed 4-fold increase of proliferation to the signal $s_1$, which is calibrated to cause at most a 4-fold increase of the proliferation rate. It has been reported that long-term survivors after bone marrow transplantation have reduced HSC counts, compared to the donors^36^. This finding suggests that HSC counts are not strictly regulated as long as mature cell counts are in the physiological range^61^. We incorporate this finding in our model by neglecting small values of the signal $s_2$. Only if the niche-related stimulation $s_2$ is larger than the systemic stimulation $s_1$, the niche-derived signal has an impact and induces a switch to fast HSC proliferation, as observed in the post-chemotherapy period, to avoid depletion of the HSC pool and to re-establish a reserve HSC population.

As in^26,62^, we assume that LSCs exhibit a constitutive activation of signaling pathways^63,64^ and, therefore, proliferate at higher rates, i.e., at the rates which healthy cells exhibit under maximal stimulation:

4 $$\begin{aligned} \widetilde{s}_{lsc}(t)~&= \left\{ \begin{array}{rcl} {s}_{max} & & {{s}_{1}(t)~ < {s}_{2}(t)~}\\ 1 & & {{s}_{1}(t)~ \ge {s}_{2}(t)~}\\ \end{array} \right. \end{aligned}$$

The LSC proliferation rate at time *t* is $p_1^l(t)={\tilde{p}}_1^l\cdot \tilde{s}_{lsc}(t)$, where the constant ${\tilde{p}}_1^l$ is estimated from the individual patient data.

This work aims to understand the HSC dynamics. Since the HSC population gives rise to all types of mature blood cells, we use the model parametrization from^26^, which accounts for the total daily output of mature cells (red, white and platelet). For simplicity we do not distinguish between different mature cell types but consider one averaged healthy cell lineage. Simulation and parameter fitting details are provided in the Section S2 of the Supplement, Supplemental Figures S2-S3 as well as Supplemental Tables S1-S3.

In Section S4 of the Supplement, we consider different extensions of the proposed model accounting for the suppression of healthy blood cell formation by paracrine signals such as IL6^65^ and for perturbations of systemic feedback signals by cytokine receptors (e.g., MPL) expressed on leukemic blasts^66^.

## Results

### The proposed model can recapitulate HSC dynamics after 7+3 chemotherapy

We have proposed a model of human HSC dynamics after 7+3 chemotherapy, Fig. 1. This model accounts for healthy and leukemic stem, progenitor and precursor cells as well as for mature blood cells and post-mitotic leukemic blasts. It incorporates competition of HSCs and LSCs in a joint stem cell niche, where LSCs can dislodge HSCs and vice versa. The HSC proliferation rate is regulated by a systemic signal which depends on the concentration of mature cells and by a micro-environmental signal, reflecting the frequency of empty niche spaces. The proliferation rate of progenitors and the number of divisions they perform before differentiation also depend on the systemic feedback signal.

**Figure 2:**
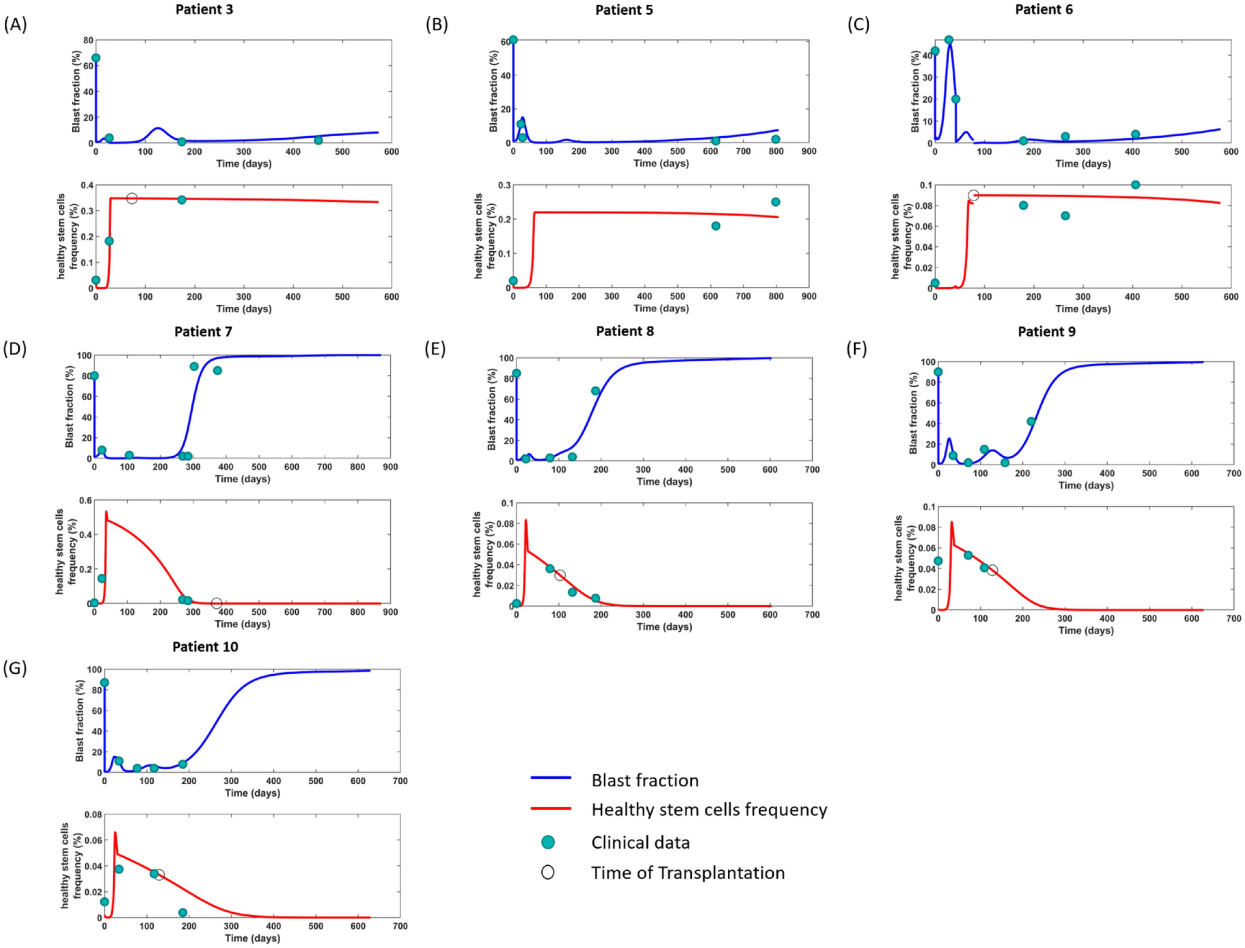
Fit of the model to clinical data from^28^. Green dots represent the clinical data; blue lines indicate the blast fraction and red lines correspond to HSCs frequency. (**A–C**) Patients achieving complete remission and remaining disease free until the end of follow up. For the patient in panel (**C**) we simulate in addition to the 7+3 scheme a high-dose 3 days AraC therapy since the blast counts have not declined under the 7+3 treatment. The AraC treatment is followed by a stem cell transplantation. We simulate the transplantation that the patient received at day 78 by filling all the empty niche space with healthy stem cells. This leads to the discontinuity in the graph showing the HSC frequency. (**D-G**) Patients relapsing during the follow-up. Based on the clincal data, all patients except Patient No. 5 received transplantation sometime after chemotherapy and the timepoint has been marked as a black circle on the time evolution of HSCs frequency (red lines). However, the niche capacity of patient No. 3, 7, 8, 9, 10 is full at the time when we simulate transplantation and there are no stem cells abundance change on the simulation results. Dynamics of post-mitotic cells are provided in Supplemental Fig. S5.

We fitted the model to example patients from the study by Wang et al.^28^ where HSCs (CD34+CD38-ALDH+ cells) were longitudinally quantified. LSC and blast properties as well as drug-induced apoptosis rates were fitted individually to the patients to reflect the high inter-individual variability of AML dynamics and disease-driving mutations. Our model can reproduce the fast HSC expansion after chemotherapy. Figure 2A–C, shows patients achieving complete remission and remaining disease free for 500 days or longer. In these patients, the HSC counts eventually stabilize at a level which is similar to that of healthy individuals^28^.

**Figure 3:**
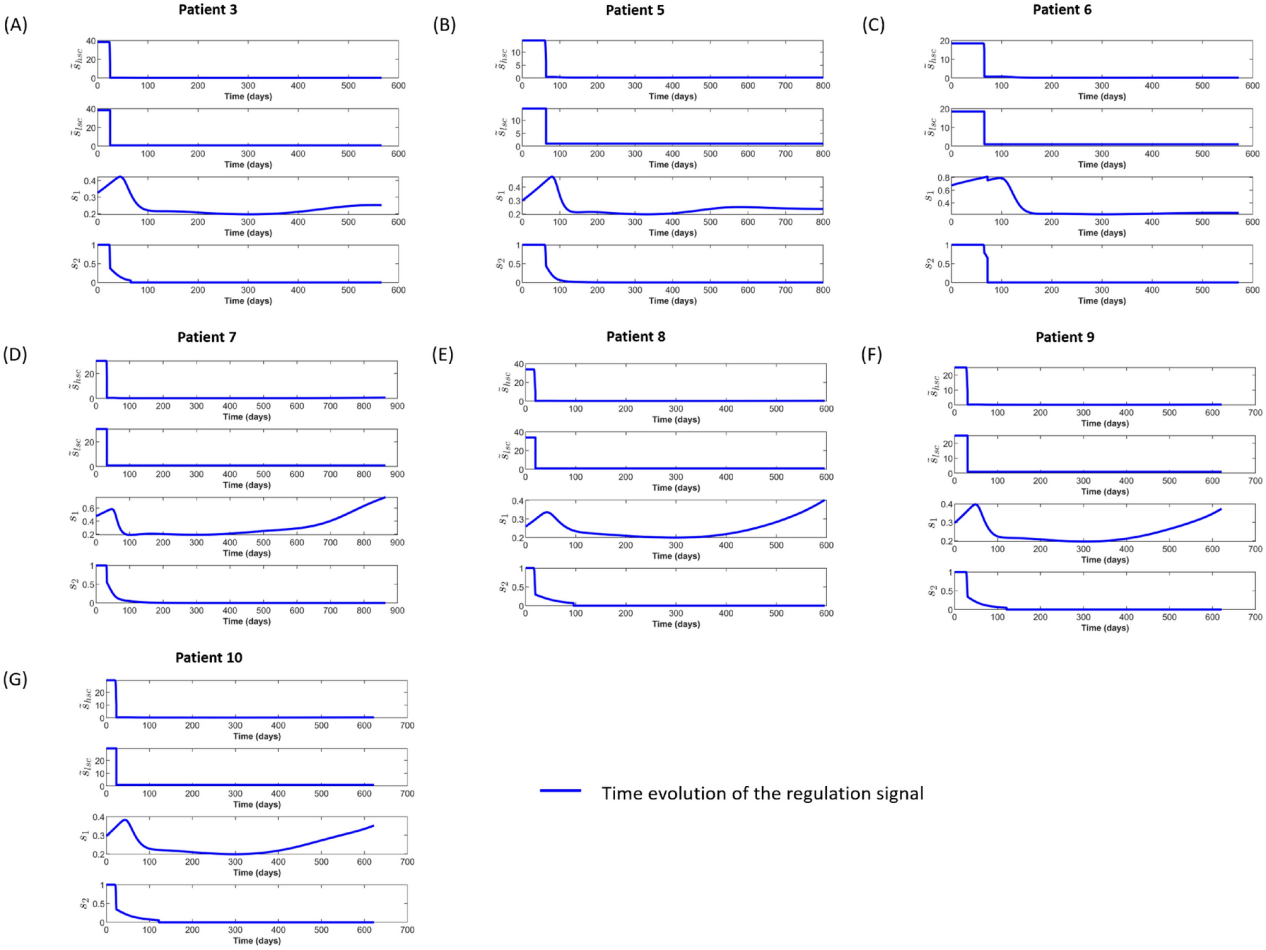
Simulation results of the time evolution of each feedback signal. On each panel, ${\tilde{s}}_{hsc}$ describes the change of the proliferation rate of HSCs and ${\tilde{s}}_{lsc}$ of LSCs. $s_1$ is the systemic feedback signal that depends on the abundance of healthy mature blood cells. $s_2$ is the niche-related feedback signal depending on the number of empty niche spaces. Both signal are integrated into the cellular responses denoted as ${\tilde{s}}_{hsc}$ and ${\tilde{s}}_{lsc}$.

Figure 2D–G show relapsing patients. These patients exhibit a transient decrease of blasts frequencies which is accompanied by a transient increase of HSC abundance. The increase of HSCs occurs since the LSC burden in the stem cell niche has been reduced and proliferative signals trigger stem cell expansion. Upon growth of the LSC burden, HSCs are out-competed from the niche and their counts decrease. The fitted parameters are summarized in Supplemental Table S3. Figure 3 shows the time evolution of the feedback signals for each patient. We observe that the signal is maximal in the days after the start of chemotherapy and exhibits a pronounced decline thereafter. These signal dynamics can faithfully capture the time evolution of HSCs observed in the clinical data, especially the drastically increased proliferation rate in the immediate aftermath of transplantation. If we omit the niche-derived feedback signal the model does not exhibit realistic cell dynamics, see Supplemental Figure S4 and Supplement S3.

### Impact of cell properties on treatment outcome

**Figure 4:**
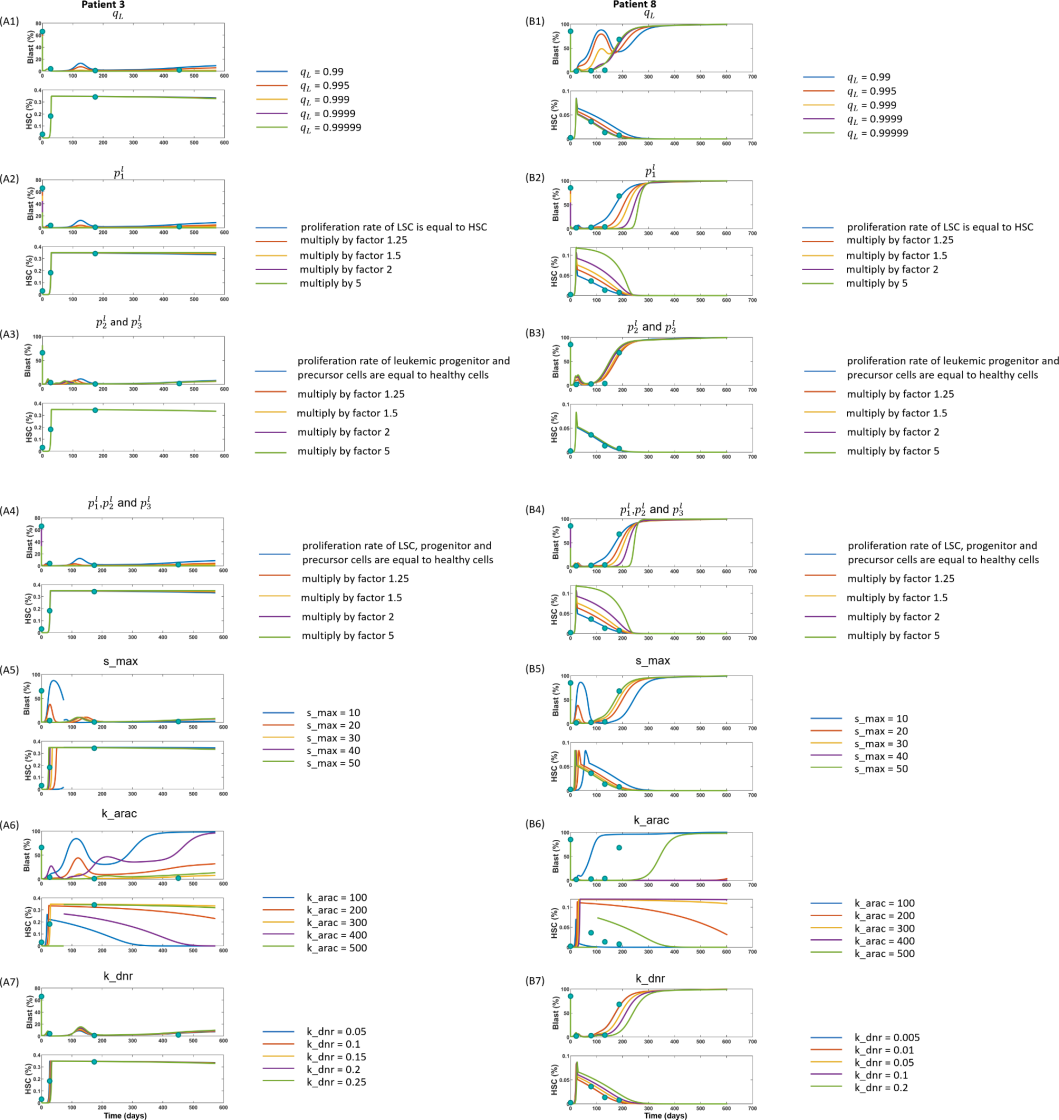
Impact of leukemic cell and drug parameters on treatment response. We consider the patients from Fig. 2A, E). We perturb the HSC dislodgement probability $q_{L}$ (**A1, B1**), the leukemic stem, progenitor and precursor proliferation rates $p^{l}_{1}, p^{l}_{2}, p^{l}_{3}$ (**A2-A4**,** B2-B4**), the increase of stem cell proliferation rates in response to the feedback signal $s_{max}$ (**A5, B5**), and the drug effect parameters $k_{arac}$ and $k_{dnr}$ (A6-A7, B6-B7). $s_{max}$ corresponds to the maximal possible increase of the stem cells proliferation rate in response to the niche-derived feedback signal. Panels (**A1–A7**) use the parameter set and clinical data from Fig. 2A (clinical data from patient No. 3) and panel (**B1–B7**) use the parameter set and clinical data from Fig. 2E (clinical data from patient No. 8). A stem cell transplantation is simulated at the exact time when the patient received it according to the medical records. We simulate the transplantation by immediately refilling all empty niches with HSCs. This simplification can lead to a discontinuity of the graph which shows the time evolution of HSC frequency.

We use the fitted model to study how leukemic cell and drug properties impact on the treatment response. We choose two example patients, one patient (patient 3, Fig. 4A) achieving long term remission and one patient (patient 8, Fig. 4 (B)) exhibiting an early relapse. The simulations allow us to infer how the disease course of the considered patients might have changed if the respective parameters assumed different values. In our model, HSCs and LSCs compete for a joint stem cell niche. This implies that offspring originating from the division of LSCs can dislodge HSCs from the niche and vice versa. The probability by which LSCs dislodge HSCs is referred to as *HSC dislodgement probability, *$q_L$. In Fig. 4A1, B1 we perturb the HSC dislodgement probability $q_L$. In panel (A1) we observe a mild temporary increase of the blast fraction between day 100 and day 200. The reason for this is the proliferation and differentiation of residual leukemic cells, which have survived chemotherapy and which sense a strong proliferative signal in the aftermath of the treatment. The lower $q_L$, the higher the probability that LSCs differentiate and the more pronounced is the temporary increase. When the proliferative signal normalizes to equilibrium values the production of leukemic progenitors significantly drops and the blast count declines. For values of $q_L$ above 0.9999 no temporary increase is observed, since residual LSCs rather self-renenew instead of giving rise to blasts^26^. This might correspond to what is clinically observed in most patients. The same tendency is observed in panel (B1). Next, we perturbed the leukemic stem cell proliferation rate $p_1^l$ (Fig. 4A2, B2). We observe that a high LSC proliferation rate leads to an efficient eradication of LSCs and a later relapse, since the therapy preferentially targets mitotic cells. As panels (A3)–(A4) and (B3)–(B4) demonstrate, leukemic progenitor and precursor proliferation rates $p_2^l$ and $p_3^l$ have little impact on the therapy outcome. Panels (A5) and (B5) investigate how the up-regulation of the stem cell proliferation rate affects the response to chemotherapy. The parameter $s_{max}$ quantifies by how many times the niche-derived feedback signal can increase the HSC and LSC proliferation rates. For small values of $s_{max}$ we observe a temporary increase of the blast counts between days 0 and 50. This increase is triggered by the residual LSCs. As soon as HSC counts have recovered, the blast counts decline again. In panels (A6)–(A7) and (B6)–(B7) we investigate how parameters of chemotherapy impact on cell dynamics. The higher $k_{arac}$ and $k_{dnr}$, the more cells are killed per unit of time by AraC and DNR, respectively. We observe that a reduction of the AraC effect leads to early relapses and prohibits complete remission. The AraC effect depends on the proliferation rate and therefore differently affects stem and progenitor cells. If leukemic progenitors and precursors survive chemotherapy, they are flushed out over time due to differentiation. This may lead to non-monotonous blast dynamics. In summary, we observe that the HSC dislodgement probability, the leukemic cell proliferation rate and the effect of the chemotherapeutic drugs impact on the time to relapse. According to our simulations a resistance to AraC has more pronounced effects compared to a resistance to DNR. Properties of leukemic progenitor cells have no impact.

### G-CSF priming improves outcome in model simulations

**Figure 5:**
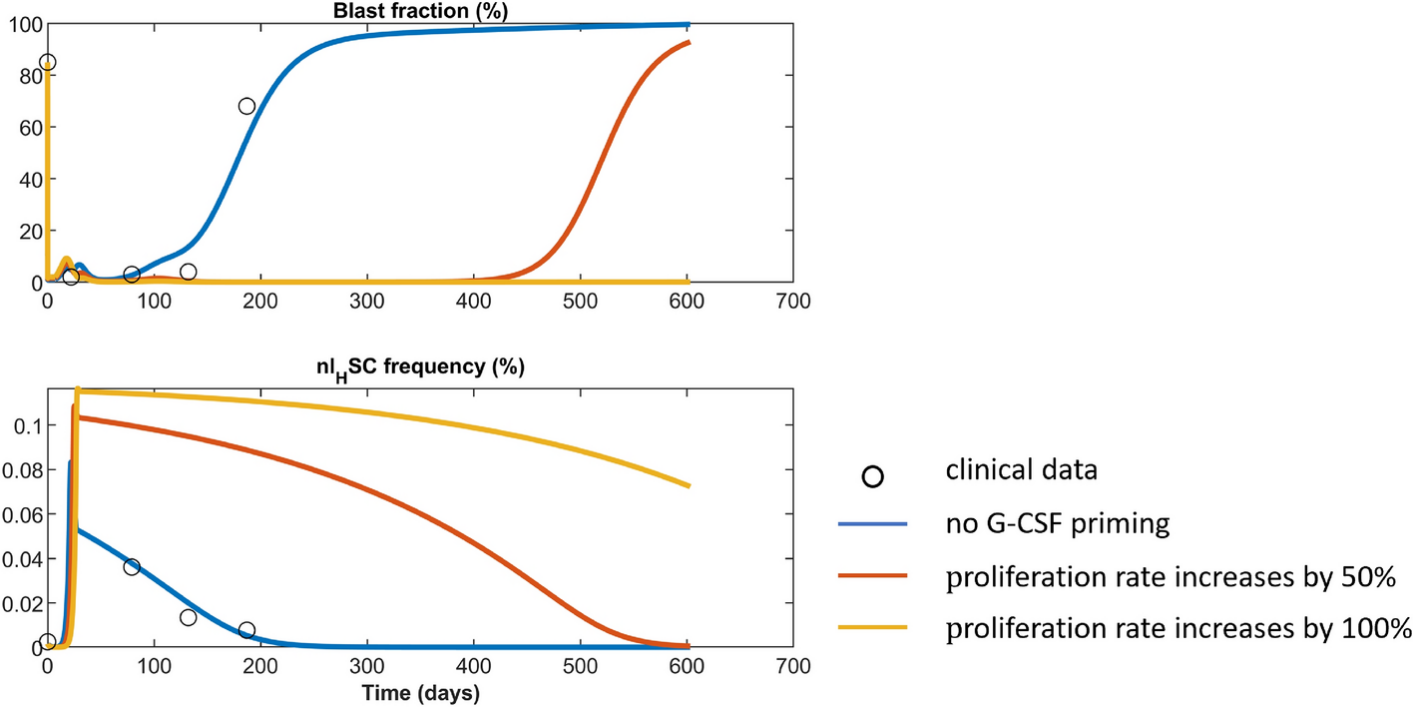
Simulation of priming with G-CSF during induction therapy. The black circles correspond to clinical data of patient No. 8 from^28^. The blue line shows the fit to the clinical data and is identical to Fig. 2E. The red line shows treatment response after G-CSF priming which increase all healthy and leukemic stem, progenitor, and precursor cells proliferation rates by 50%. The yellow line shows the model simulations for increasing the proliferation rates by 100%.

Clinical trials suggest that priming with granulocyte colony-stimulating factor (G-CSF) might be effective in AML treatment^67,68^. G-CSF modulates the proliferation rate of immature hematopoietic cells^69^. To simulate how cytokine priming could affect the therapy response, we increase the proliferation rate of all mitotic hematopoietic and leukemic cell types during the administration of the 7+3 scheme. We consider two scenarios. In the first scenario (red line in Fig. 5), the G-CSF priming increases the proliferation rate by $50\%$. In the second scenario (yellow line in Fig. 5), G-CSF priming increases the proliferation by $100\%$. We observe that G-CSF priming in our model substantially improves treatment outcome. This is in line with trial results reporting a higher disease-free survival after G-CSF priming^67^.

### Decrease of HSC counts could be used as an indication for salvage therapy

In agreement with biological and clinical data, the relapse in the model simulation is triggered by residual LSCs. Due to the slow growth kinetics it can take months to years until the blast fraction surmounts 5% and thus fulfills the criterion of a hematologic relapse. Especially if no MRD markers exist, what is the case for a significant proportion of the patients^70^, the late detection of relapse might reduce patient survival. Wang et al.^28^ have proposed to use the decline of HSC as an early marker of relapse.

**Figure 6:**
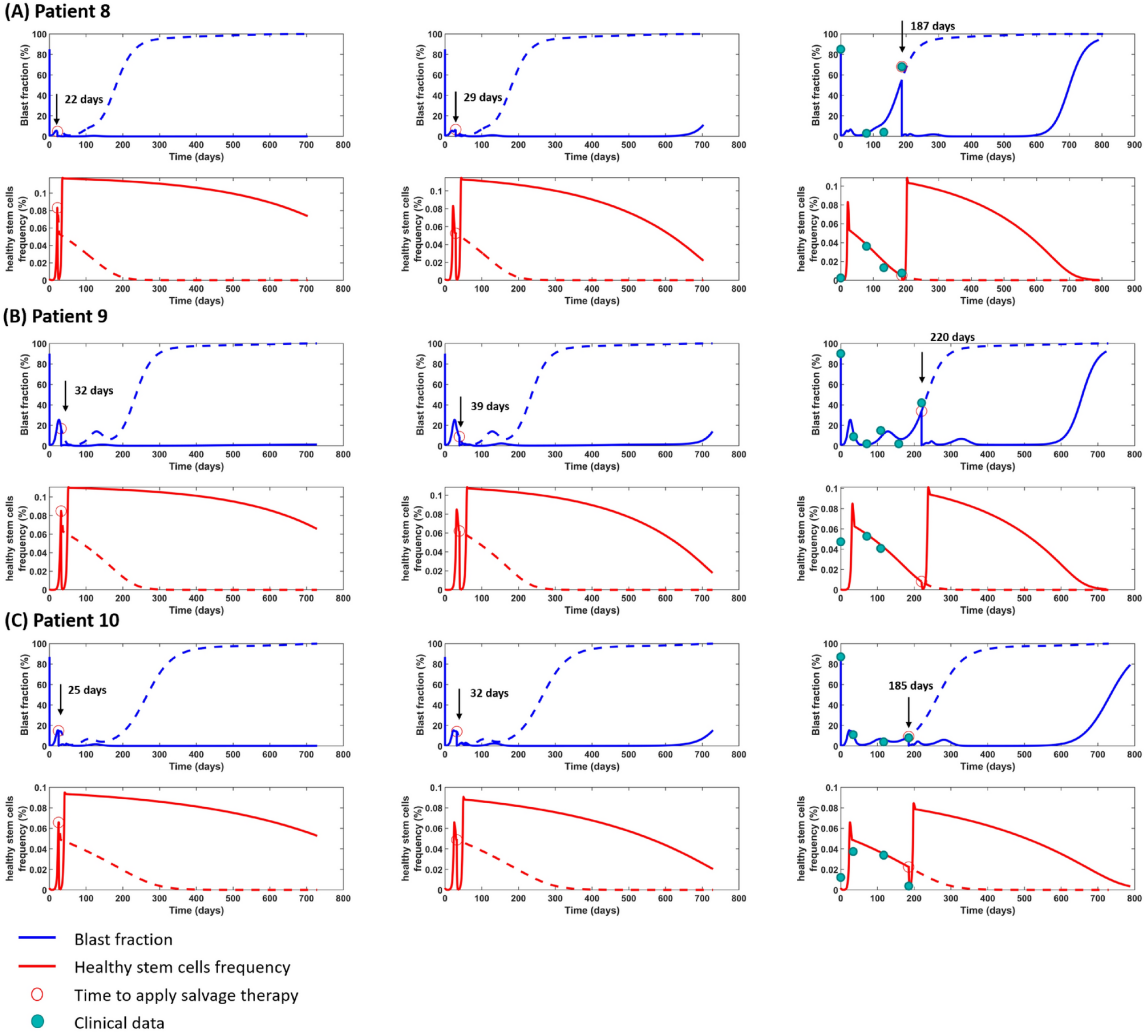
Comparison of different indications for salvage therapy. We consider the patients from Fig. 2E–G. The dashed lines indicate the disease evolution in absence of salvage therapy. They are identical to the simulations in Fig. 2E–G. In the left column therapy is initiated at the time, when the HSC counts start to decline. In the second column therapy is initiated one week after HSC counts started to decline. In the third column salvage therapy is initiated at the time of the first clinical measurement reporting more than 5% blasts. Red circles mark the time point where salvage therapy starts. Simulation results in Panel (**A**) using same parameters set and clinical data as in Fig. 2E, Panel (**B**) corresponds to Fig.2F, and Panel (**C**) is corresponds to Fig. 2G. We observe that in the model simulations the timing of salvage therapy is crucial. The time evolution of post-mitotic cells is provided in Supplemental Fig. S6.

We use our *in silico* model to simulate how the outcome after salvage therapy would change if the decline of HSCs had been used as an treatment indication. In our simulations we start the salvage therapy when the HSC counts start to decline. Fig. 6 shows example simulations for patients in Fig. 2E–G. We observe that in the simulations the outcome improves if the decline of HSCs is used as an indication for salvage therapy.

### Chemotherapy induced side-effects leading to poor treatment outcomes

#### Even mild chemo-protective effects of the niche may reduce patient outcome

**Figure 7:**
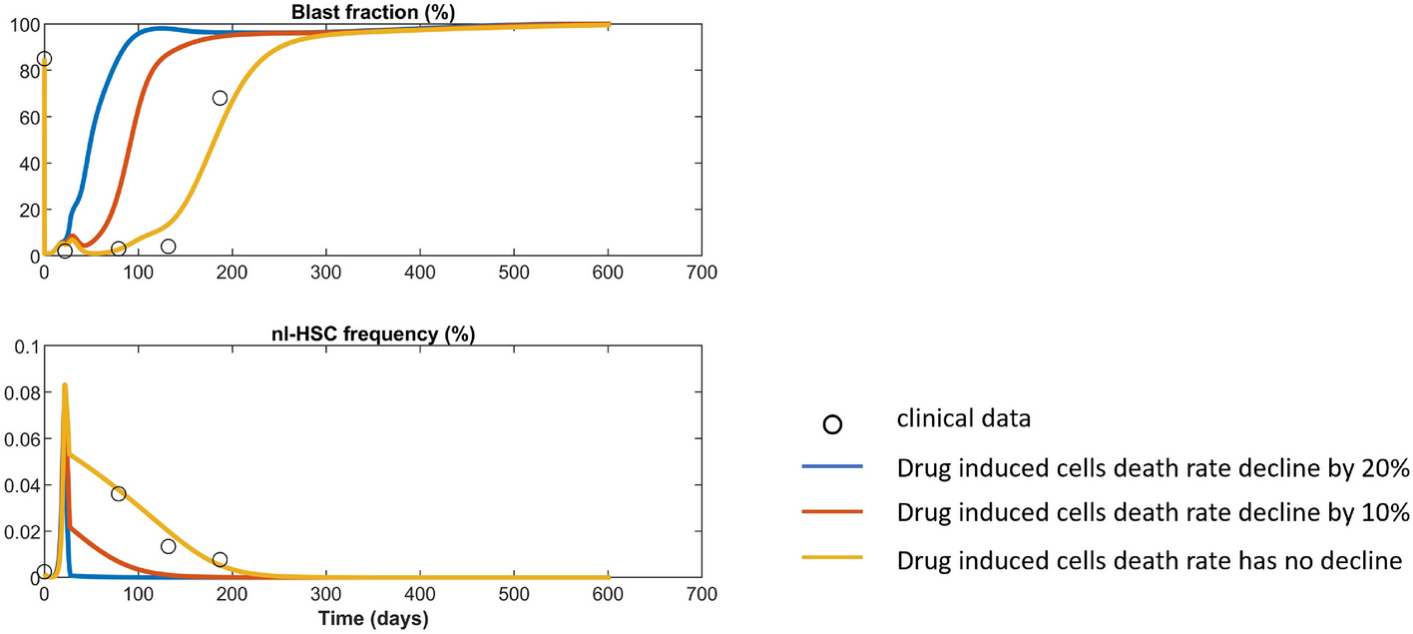
Simulation of chemotherapy resistance from bone marrow niche. The black circles correspond to clinical data of patient No. 8 from^28^. The yellow line shows that fit to the clinical data and is identical to Fig. 2E. The red line shows the simulation results when AraC and DNR drugs induced stem cells death rate decline by 10% and the blue line shows the simulation results when they decline by 20%.

There is evidence that the bone marrow niche can increase resistances to chemotherapy^71,72^. We simulate this by reducing the drug induced death rates $k_{arac}$ and $k_{dnr}$ acting on HSCs and LSCs. We run simulations for reductions by $10\%$ (red line in Fig. 7) and by $20\%$ (blue line in Fig. 7). As expected, we observe that a niche-mediated resistance to therapy, even if relatively mild, has a considerable impact on the therapy outcome. In our example simulation a reduction of the drug effect by 10% reduces the time to relapse from about 200 days to about 100 days.

#### Therapy-related reduction of the niche capacity may lead to poor outcome

**Figure 8:**
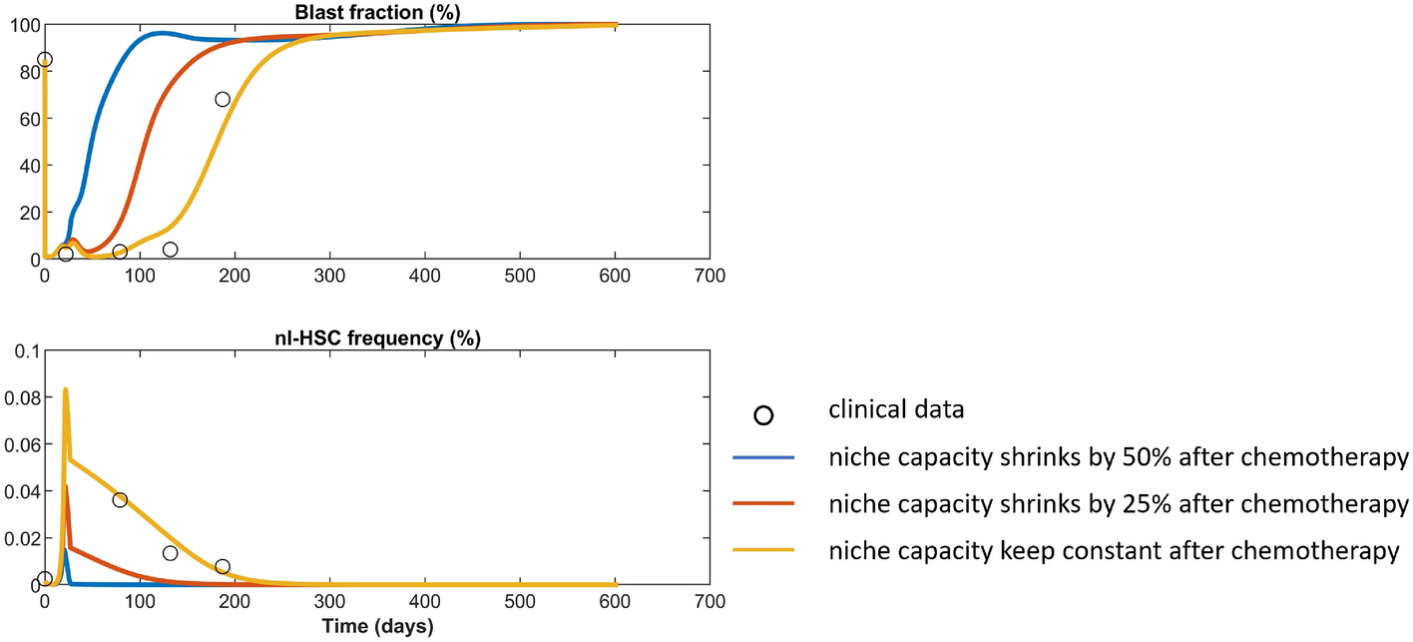
Simulation of possible niche damage after chemotherapy. The black circles correspond to clinical data of patient No. 8 from^28^. The yellow line shows that fit to the clinical data and is identical to Fig. 2E. The red line shows the simulation results when niche capacity shrinks by 25% after chemotherapy and the blue line shows the simulation results when it shrinks by 50%.

There is evidence that cytotoxic therapy induces damage of the bone marrow niche^73^. We simulate this by reducing the niche capacity $K$ to 50% and 75% of its physiological size post-chemotherapy, Fig. 8. We observe that a reduction of the niche capacity leads to worse outcomes. Even if only a subset of HSCs is required to maintain steady state hematopoiesis, a smaller niche leads to a faster out-competition of healthy hematopoiesis.

#### Chemotherapy induced quiescence diminishes the efficacy of treatment

**Figure 9:**
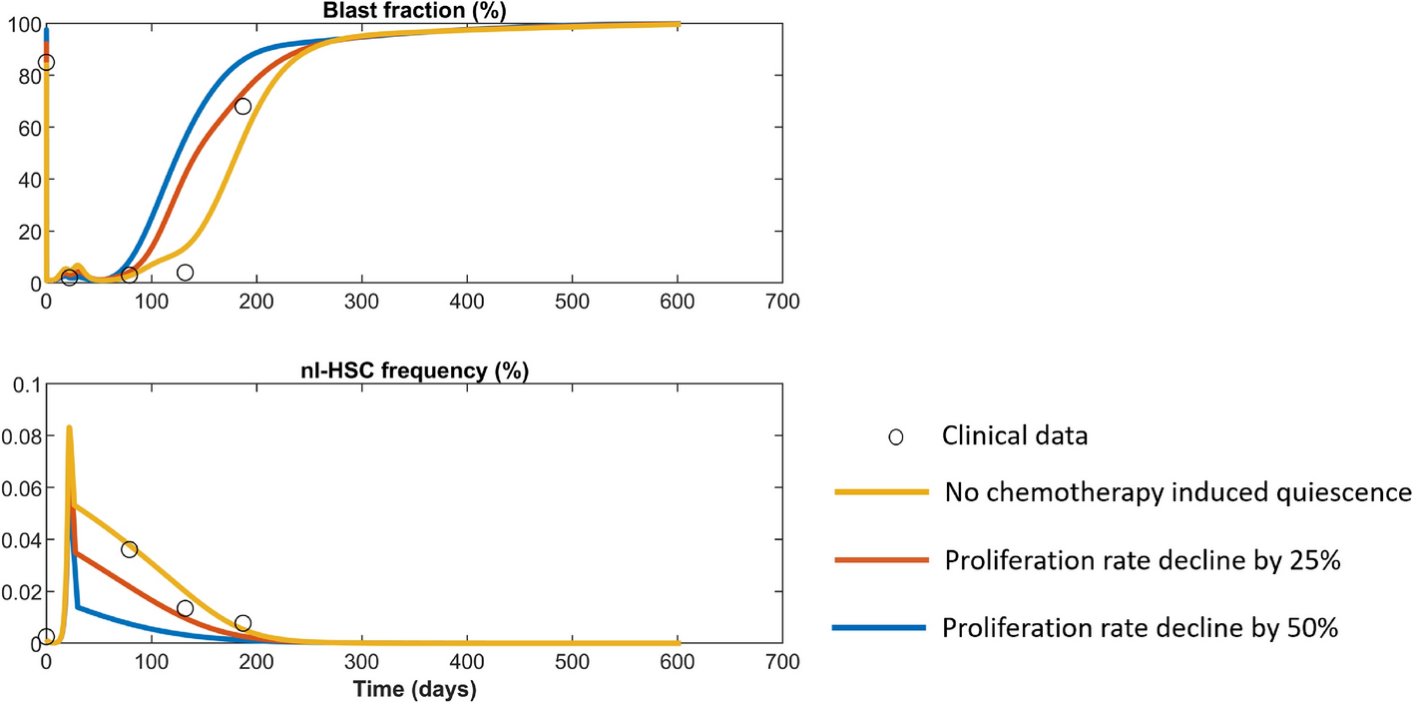
Impact of chemotherapy-induced senescence. The black circles correspond to clinical data of patient No. 8 from^28^. The yellow line shows the fit of the model without therapy-induced senescence to the clinical data and is identical to Fig. 2E. The red line shows the simulated disease evolution when the proliferation rates of all immature leukemic cells are reduced by 25% during and after chemotherapy. The blue line shows the simulation results for a reduction of leukemic cell proliferation rates by 50%.

There is evidence that AML cells can enter a quiescent (senescence-like) state which is induced by exposure chemotherapy^74^* in vitro* and *in vivo*. To simulate the chemotherapy-induced quiescence, we reduce the proliferation rate of all immature leukemic cells ($p^l_1,p^l_2,p^l_3$) by 25% (red line in Fig. 9) or 50% (blue line in Fig 9) during and after chemotherapy. In line with^74^, we observe that this results in an earlier relapse of the disease.

## Discussion

The aim of this work is to study HSC dynamics after AML remission induction chemotherapy. We extend a model of the stem cell niche to account for the effects of cytarabine and daunorubicin. To provide quantitative insights into HSC dynamics, we fit the model to data from a clinical trial which logitudinally quantified CD34+CD38-ALDH positive cells in AML patients. This cell fraction is highly enriched for HSCs and devoid of LSCs^28^.

Comparison of model simulations to data suggests the HSC proliferation rate after chemotherapy to be at least 10-fold faster than in the equilibrium state. To recapitulate this observation, we propose a feedback mechanisms which depends on the amount of empty niche spaces. We fitted the extended model to 7 patient examples from^28^. The model is in good agreement with HSC and blast dynamics of 6 out of the 7 patients (Fig. 2A–E, G). For one of the patients (Fig. 2F) the model failed to reproduce the HSC frequency at diagnosis. A possible reason for this is that the respective patient suffers from undifferentiated AML (M0), which is a rare subtype exhibiting an absence of clear lymphoid and myeloid markers^75^. The primitive phenotype of the leukemic cells might potentially interfere with the HSC quantification. Another possible explanation is that the diagnostic marrow sample is not representative. Despite the misfit at the time of diagnosis, the model is in good agreement with later dynamics.

Our model is able to recapitulate the fast increase of HSCs in patients achieving complete remission and the non-monotonous HSC dynamics in relapsing patients. The underlying mechanism is as follows: Due to the competition of HSCs and LSCs for niche spaces, the therapy-induced decline of LSCs is followed by an expansion of HSCs. When residual LSCs proliferate and start to take over the niche, HSCs are dislodged and out-competed, which leads to a measurable reduction of HSCs. As observed in patients^28^, the decline of HSCs in our simulations starts before clinically overt relapse. Therefore, HSC dynamics could serve as an indicator of relapse in patients without MRD markers^28^. Simulation of our model suggests that administration of a salvage therapy (3 days high dose cytarabine) at the time of HSC decline leads to better outcomes compared to a salvage therapy which is applied at the time of hematological relapse ( $5\%$ marrow blasts).

An important drawback of our study is the limited availability of longitudinal HSC data. It has be noted that HSC quantifications are challenging and depend on the used markers^76,77^ . Relative HSC quantification (HSCs per MNC) cannot reveal absolute HSC counts, whereas absolute quantifications (HSCs per micro liter of bone marrow) may be confounded by apsiration of blood. Another limitation comes from the neglection of stochastic effects which may play a role if cell counts are low. In addition it has to be noted, our model simplifies the effects of AraC and DNR. At the moment our model accounts only for the first 7+3 cycle and potential salvage therapies. Due to the small number of HSC measurements we have not considered consolidation therapy.

Since the focus of this work is on HSC dynamics we have not considered different subtypes of mature cells. This considerably simplifies the model and its parametrization. Instead of separate lineages we consider an averaged mature cell population that accounts for all mature cell types. It is calibrated in a way which reflects the total daily turnover of mature blood cells and thus ensures that the number of cells to which the HSC population gives rise per day assumes a realistic order of magnitude. Restricting the model to one lineage would underestimate the amount of cells produced per day. A drawback of this approach is, however, that the simulated mature cell counts are challenging to interpret.

A recent study from^65^ demonstrates that AML blasts can secrete factors such as IL6 which drive peripheral cytopenia. We have considered an extended version of our model which accounts for this finding (Section S4.2 of the Supplement). The modified model shows pronounced peripheral cytopenia but similar stem cell dynamics (Fig. S8). Another interesting finding is that the expression of MPL on AML blasts reduces systemic thrombopoietin concentrations which interferes with healthy blood cell formation^66^. As a proof of principle we have simulated this mechanism by assuming that the systemic feedback signals depend not only on healthy mature cells but also on AML blasts (Section S4.2 of the Supplement and Fig. S9). In agreement with the findings from^66^ this mechanism leads to peripheral cytopenia.

A further simplification is that our model does not account for circulating HSCs, which recent studies have shown to possess distinct characteristics compared to BM-resident HSCs^78,79^. Given that over 99% of hematopoietic stem and progenitor cells reside in the bone marrow^78^, we assume that this simplification does not significantly impact on model dynamics. This is in line with the observations that model dynamics only slightly change when we assume that dislodged HSCs do not immediately differentiate (Fig. S7). Also, our model has not considered the spatial organization of bone marrow cells studied in^80^. This requires more complex models, which can be implemented in the future work.

Our model allows to investigate various scenarios discussed in the literature, such as G-CSF priming before chemotherapy, niche-mediated therapy resistance, and therapy-related impairment of niche function. The outcomes of these simulations may offer quantitative insights into the intricate dynamics of AML progression, contributing to a nuanced understanding of potential therapeutic interventions within the framework of computational and systems oncology.

## Electronic supplementary material

Below is the link to the electronic supplementary material.

Supplementary Information.

## Data Availability

All data generated or analysed during this study are included in this published article and its supplementary information files.
