# Supplementary material for: Modelling post-chemotherapy stem cell dynamics in the bone marrow niche of AML patients
Source: Sci Rep. 2024 Oct 23;14:25060. doi: 10.1038/s41598-024-75429-7 (PMC11500015; doi:10.1038/s41598-024-75429-7)
Supplement: Supplementary file 1 — Supplementary Information. [file 41598_2024_75429_MOESM1_ESM.pdf]

# Supplemental Materials

## S1 Mathematical Model of AML cell dynamics

This section describes the model of the stem cell niche which is summarized in Fig.S1. The derivation follows references[1,2]. We account for two cell lineages, one healthy and one leukemic. The leukemic cell lineage is comprised of leukemic stem cell (LSC), leukemic progenitor cells (LProC), leukemic precursor cells (LPreC), and post-mitotic leukemic blasts. Analogously, the healthy cell lineage consists of hematopoietic stem cells (HSC), hematopoietic progenitor cells (HProC), hematopoietic precursor cells (HPreC), and post-mitotic mature blood cells. In agreement with recent experimental results[3] suggesting the invasion of the hematopoietic stem cell niche by LSC, we assume that HSC and LSC compete for spaces in a joined bone marrow niche, which is required to maintain the stem cell state.

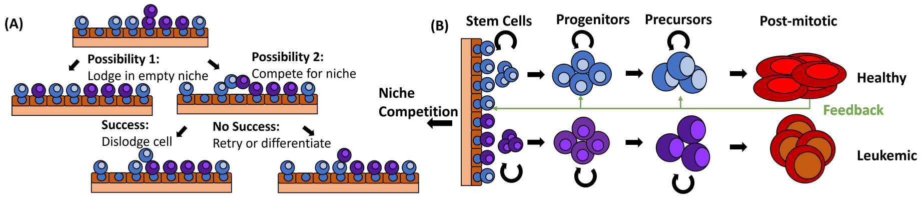


**Figure S1.** Model of acute myeloid leukemia from[1], which illustrates the stem cells competition inside bone marrow niche and feedback signal regulation of hematopoietic cells growth.

### S1.1 Mechanistic model of the human HSC niche

We denote the number of HSCs per kilo of body weight at time $t$ as $c_1(t)$ and that of LSCs as $l_1(t)$. The proliferation rates are referred to as $p_1^c(t)$ and $p_1^l(t)$, respectively. The mechanism of niche competition is illustrated in Fig. S1. The capacity of the bone marrow niche is denoted as $K$. Upon division of a LSC, two progeny emerge, one of which occupies the niche space of its parent. The other attempts to access a random niche space. If the randomly chosen niche space is empty (the probability for that scenario equals $p_e(t) = \frac{K-c_1(t)-l_1(t)}{K}$) the cell occupies it and preserves its stemness. If the niche space is occupied by a HSC (the probability for that scenario equals $p_c(t) = \frac{c_1(t)}{K}$), the HSC can be dislodged with a certain probability, referred to as HSC dislodgement probability ($q_L$). If the accessed niche space is occupied by a LSC (the probability for that scenario equals $p_l(t) = \frac{l_1(t)}{K}$), the space is not conquered by the daughter cell. Analogous processes happen after the division of a HSC. We denote the probability by which a HSC dislodges a LSC as LSC dislodgement probability $q_H = 1 - q_L$. Cells dislodged from the niche or cells finding no niche space within $n$ attempts are assumed to differentiate into progenitors. We denote the chemotherapy-induced death rate of HSCs and LSCs as $d_1^{c,chemo}(t)$ and $d_1^{l,chemo}(t)$.

Consequently, the time evolution of LSC abundance is described by equation S1 and that of HSCs by equation S3. The flux from LSC to LProC ($v_1^l$) is given by equation S4 and equation S5 is the flux from HSC to HProC ($v_1^c$). For the sake of convenience we omit the variable $t$ in the notation.

$$\frac{d}{dt}l_1 = \underbrace{-p_1^l l_1}_{to\ mitosis} + \underbrace{(1-(1-p_e-p_c q_L)^n)\,p_1^l l_1}_{to\ empty\ space\ or\ dislodging\ HSC} + \underbrace{p_1^l l_1}_{to\ parents'\ space} \underbrace{-(1-(1-p_e-p_l q_H)^n)\,\frac{p_l q_H}{p_e+p_l q_H}\,p_1^c c_1}_{dislodged\ by\ HSC} - d_1^{l,chemo} l_1 \quad \text{(S1)}$$

$$\frac{d}{dt}l_1 = \left(1-\left(1-\frac{K-c_1-l_1}{K}-q_L\frac{c_1}{K}\right)^n\right)p_1^l l_1 - \left(1-\left(1-\frac{K-c_1-l_1}{K}-q_H\frac{l_1}{K}\right)^n\right)\frac{l_1 q_H}{K-l_1-c_1+q_H l_1}p_1^c c_1 - d_1^{l,chemo} l_1 \quad \text{(S2)}$$

$$\frac{d}{dt}c_1 = \left(1-\left(1-\frac{K-c_1-l_1}{K}-q_H\frac{l_1}{K}\right)^n\right)p_1^c c_1 - \left(1-\left(1-\frac{K-c_1-l_1}{K}-q_L\frac{c_1}{K}\right)^n\right)\frac{c_1 q_L}{K-l_1-c_1+q_L c_1}p_1^l l_1 - d_1^{c,chemo} c_1$$

(S3)

$$v_1^l = \left(1 - \frac{K - c_1 - l_1}{K} - q_L \frac{c_1}{K}\right)^n p_1^l l_1 + \left(1 - \left(1 - \frac{K - c_1 - l_1}{K} - q_H \frac{l_1}{K}\right)^n\right) \frac{l_1 q_H}{K - l_1 - c_1 + q_H l_1} p_1^c c_1 \tag{S4}$$

$$v_1^c = \left(1 - \frac{K - c_1 - l_1}{K} - q_H \frac{l_1}{K}\right)^n p_1^c c_1 + \left(1 - \left(1 - \frac{K - c_1 - l_1}{K} - q_L \frac{c_1}{K}\right)^n\right) \frac{c_1 q_L}{K - l_1 - c_1 + q_L c_1} p_1^l l_1 \tag{S5}$$

### S1.2 Mechanistic model of progenitor and precursor cell dynamics

In agreement with biological observations[4], the model assumes that progenitor and precursor cells can only perform a limited number of divisions before further differentiation. The maximal number of divisions progenitor cells can perform before further differentiation is denoted by $n_1$ and that of precursors by $n_2$.

To account for the number of cell divisions, the leukemic progenitor compartment has been divided in $(n_1+1)$ sub-compartments, e.g. $l_{2,0}, \cdots, l_{2,i}, \cdots, l_{2,n_1}, (1 \le i \le n_1)$. Hence, $l_{2,i}$ denotes the sub-compartment of leukemic progenitors that have performed $i$ divisions since they have entered the progenitor state. Analogously, the healthy progenitor compartment consists of $(n_1+1)$ sub-compartments $(c_{2,0}, \cdots, c_{2,i}, \cdots, c_{2,n_1}, (1 \le i \le n_1))$; the leukemic precursor compartment consists of $(n_2+1)$ sub-compartments $(l_{3,0}, \cdots, l_{3,i}, \cdots, l_{3,n_2}, (1 \le i \le n_2))$; and the healthy precursor compartment consists of $(n_2+1)$ sub-compartments $(c_{3,0}, \cdots, c_{3,i}, \cdots, c_{3,n_2}, (1 \le i \le n_2))$.

In the leukemic progenitor compartment, $p_2^l(t) l_{2,i}(t)$ cells enter division per unit of time, where $p_2^l(t)$ refers to the division rate of LProC at time $t$. Analogously, the division rates of HProC, LPreC and HPreC are denoted as $p_2^c(t)$, $p_3^l(t)$, and $p_3^c(t)$. The quantities $a_2^l(t)$ and $a_2^c(t)$ are referred to as the fractions of self-renewal for leukemic and hematopoietic progenitor cells. By $a_3^l(t)$ and $a_3^c(t)$ we denote the respective fractions of self-renewal for leukemic and hematopoietic precursor cells. The fraction of self-renewal corresponds to the probability that an offspring arising from division adopts the same fate as its parent cell. The therapy induced death rates are denoted as $d_2^{l,chemo}$ and $d_2^{c,chemo}$ for the progenitors and as $d_3^{l,chemo}$ and $d_3^{c,chemo}$ for the precursors. The population dynamics of the respective cell types are given by:

$$\begin{aligned}
&\frac{d}{dt} l_{2,0} = v_1^l - p_2^l l_{2,0} - d_2^{l,chemo} l_{2,0},\\
&\frac{d}{dt} l_{2,i} = 2a_2^l p_2^l l_{2,i-1} - p_2^l l_{2,i} - d_2^{l,chemo} l_{2,i}, (1 \le i \le n_1)
\end{aligned} \tag{S6}$$

$$\begin{aligned}
&\frac{d}{dt} c_{2,0} = v_1^c - p_2^c c_{2,0} - d_2^{c,chemo} c_{2,0},\\
&\frac{d}{dt} c_{2,i} = 2a_2^c p_2^c c_{2,i-1} - p_2^c c_{2,i} - d_2^{c,chemo} c_{2,i}, (1 \le i \le n_1)
\end{aligned} \tag{S7}$$

$$\begin{aligned}
&\frac{d}{dt} l_{3,0} = 2p_2^c c_{2,n_1} + 2(1-a_2^c) p_2^c \sum_{i=0}^{n_1-1} c_{2,i} - p_3^l l_{3,0} - d_3^{l,chemo} l_{3,0},\\
&\frac{d}{dt} l_{3,i} = 2a_3^l p_3^l l_{3,i-1} - p_3^l l_{3,i} - d_3^{l,chemo} l_{3,i}, (1 \le i \le n_2)
\end{aligned} \tag{S8}$$

$$\begin{aligned}
&\frac{d}{dt} c_{3,0} = 2p_2^c c_{2,n_1} + 2(1-a_2^c) p_2^c \sum_{i=0}^{n_1-1} c_{2,i} - p_3^c c_{3,0} - d_3^{c,chemo} c_{3,0},\\
&\frac{d}{dt} c_{3,i} = 2a_3^c p_3^c c_{3,i-1} - p_3^c c_{3,i} - d_3^{c,chemo} c_{3,i}, (1 \le i \le n_2)
\end{aligned} \tag{S9}$$

The mature blood cells and post-mitotic leukemic blasts do not divide anymore, and they are eliminated at the constant rates $d_4^l$ and $d_4^c$. Furthermore, they are affected by the therapy-induced death rates $d_4^{l,chemo}$ and $d_4^{c,chemo}$. Equation S10 describes the time evolution of the post-mitotic leukemic blasts $l_4(t)$ and equation S11 describes the time evolution of mature blood cells $c_4(t)$.

$$\frac{d}{dt} l_4 = 2p_3^l l_{3,n_2} + 2(1-a_3^l) p_3^l \sum_{i=0}^{n_2-1} l_{3,i} - d_4^l l_4 - d_4^{l,chemo} l_4 \tag{S10}$$

$$\frac{d}{dt}c_4 = 2p_3^c c_{3,n_2} + 2(1-a_3^c)p_3^c \sum_{i=0}^{n_2-1} c_{3,i} - d_4^c c_4 - d_4^{c,chemo} c_4 \tag{S11}$$

## S1.3 Feedback signal regulation

The signals governing stem cell dynamics have been derived in the Methods Section. We set:

$$s_1(t) = \frac{1}{1+k_p c_4(t)} \tag{S12}$$

$$s_2(t) = \left(\frac{K - c_1(t) - l_1(t)}{1+K-c_1(t)-l_1(t)}\right) / \left(\frac{K}{1+K}\right) \tag{S13}$$

$$\widetilde{s}_{hsc}(t) = \begin{cases} s_{max} \cdot s_2(t) & s_1(t) < s_2(t) \\ s_1(t) & s_1(t) \geq s_2(t) \end{cases}, \tag{S14}$$

$$\widetilde{s}_{lsc}(t) = \begin{cases} s_{max} & s_1(t) < s_2(t) \\ 1 & s_1(t) \geq s_2(t) \end{cases} \tag{S15}$$

$$p_1^c(t) = \tilde{p}_1^c \cdot \tilde{s}_{hsc}(t) \tag{S16}$$

$$p_1^l(t) = \tilde{p}_1^l \cdot \tilde{s}_{lsc}(t) \tag{S17}$$

For the healthy progenitor proliferation and self-renewal, we set:

$$p_2^c(t) = \tilde{p}_2^c \cdot s_1(t) \tag{S18}$$

$$s_a(t) = \frac{1}{1+k_a c_4(t)} \tag{S19}$$

$$a_2^c(t) = \tilde{a}_2^c s_a(t) \tag{S20}$$

$$a_3^c(t) = \tilde{a}_3^c s_a(t). \tag{S21}$$

The parameters $k_a, \tilde{p}_1^c, \tilde{p}_1^l, \tilde{p}_2^c, \tilde{a}_2^c, \tilde{a}_3^c$ are positive constants, as probabilities $\tilde{a}_2^c, \tilde{a}_3^c$ are between zero and one.

Since precursors proliferate already fast under equilibrium conditions, we assume that their proliferation rates are not regulated. We assume that leukemic cells carry mutations which lead to a constitutive activation of signaling pathways[5,6] for proliferation and self-renewal. Therefore, leukemic progenitor and precursor cell properties are independent of feedback signals. Parameters for healthy cells are taken from[1].

## S1.4 Cell dynamics during chemotherapy

During the initial three days of chemotherapy AraC and DNR are administered. Therefore, we set:

$$d_1^{l,chemo} = k_{arac} p_1^l + k_{dnr} \tag{S22}$$

$$d_1^{c,chemo} = k_{arac} p_1^c + k_{dnr} \tag{S23}$$

$$d_2^{l,chemo} = k_{arac} p_2^l + k_{dnr} \tag{S24}$$

$$d_2^{c,chemo} = k_{arac} p_2^c + k_{dnr} \tag{S25}$$

$$d_3^{l,chemo} = k_{arac} p_3^l + k_{dnr} \tag{S26}$$

$$d_3^{c,chemo} = k_{arac} p_3^c + k_{dnr} \tag{S27}$$

$$d_4^{l,chemo} = k_{dnr} \tag{S28}$$

$$d_4^{c,chemo} = k_{dnr} \tag{S29}$$

From day 4 to 7, where only AraC is administered, we set :

$$d_1^{l,chemo} = k_{arac} p_1^l \tag{S30}$$
$$d_1^{c,chemo} = k_{arac} p_1^c \tag{S31}$$
$$d_2^{l,chemo} = k_{arac} p_2^l \tag{S32}$$
$$d_2^{c,chemo} = k_{arac} p_2^c \tag{S33}$$
$$d_3^{l,chemo} = k_{arac} p_3^l \tag{S34}$$
$$d_3^{c,chemo} = k_{arac} p_3^c \tag{S35}$$
$$d_4^{l,chemo} = 0 \tag{S36}$$
$$d_4^{c,chemo} = 0 \tag{S37}$$

Before the start and after the end of chemotherapy all drug-induced death rates equal zero. As initial conditions, we use the cell population sizes at the starting point of the therapy.

To account for the specific administration intervals of the two drugs, we adopt a sequential simulation approach using model versions that incorporate no drugs (corresponding to the pre- and post-treatment phases), both drugs (corresponding to the three initial days of therapy) and only AraC (corresponding to days 4 to 7 of therapy). Whenever the cell abundance diminishes to a level below 1 cell per kg body weight, we pragmatically set this count to 0. This approach has been used previously[7,8] and accounts for the fact that ODE models exhibit at most an exponential decline and cannot account for the total eradication of a cell population.

## S2 Simulation, Sensitivity Analysis and Fitting

### S2.1 Simulation

ODEs are solved using the function ode45 from MATLAB R2022a. First, we simulate AML disease dynamics in absence of therapy. We simulate the pre-clinical phase of AML starting from the healthy equilibrium state, where $c_1 = 9.8 \times 10^6, c_2 = 2.34 \times 10^7, c_3 = 4.03 \times 10^9, c_4 = 3.26 \times 10^{11} (unit : 1/kg\ body\ weight)$ supplemented by one LSC per kg of body weight. We simulate AML progression until the blast fraction in the model and if possible also the HSC frequency match the data at diagnosis. Chemotherapy is simulated as described in section S1.4. If a patient received a bone marrow transplantation, we model this by refilling the empty niche space with HSCs.

### S2.2 Sensitivity Analysis

To quantify the impact of model parameters on the dynamics of the system, we perform a sensitivity analysis. The time evolution of the blast fraction and HSC abundance are the most important outputs that we want to analyze. We use a one-at-a-time (OAT) technique[9], i.e., we change only one parameter at a time and keep the other fixed. This is one approach to analyze parameter sensitivity of the model locally to streamline clinical data fitting. To trigger leukemia, we set the HSC-dislodgement probability to $q_L = 0.99$. In the baseline case all other leukemic cell parameters are identical to the respective healthy cell parameters. The baseline of the HSC dislodgement probability $q_L = 0.99$ is chosen from[10]. Proliferation rates are multiplicative parameters, we test the sensitivity by multiplying by 125% and 150%. Analogously, HSC dislodgment probability and self-renewal fractions are additive parameters, we test them by adding 0.0025 and 0.005 to make sure they remain below 1. During the analysis, other parameters' values fulfill: $k_{cyt} = 200, k_{dnr} = 0.1, s_{max} = 20, d_l = 0.01$. The therapy is assumed to start when the blast fraction equals 60%.

The results of the sensitivity analysis are shown in Figure S2. We observe that the blast fraction and HSC abundance are more sensitive to changes of the LSC parameters $\tilde{p}_1^l$ and $q_L$ compared to changes of progenitor/precursor proliferation rates ($\tilde{p}_2^l$,$\tilde{p}_3^l$) and self-renewal fractions ($\tilde{a}_2^l$,$\tilde{a}_3^l$). This finding is in line with[1]. Based on the local sensitivity analysis, proliferation rates and self-renewal fractions of leukemic progenitor ($\tilde{p}_2^l$,$\tilde{a}_2^l$) and precursor cells ($\tilde{p}_3^l$,$\tilde{a}_3^l$) can be fixed for the parameter fitting, e.g. set equal to the value of the respective healthy cell parameters in order to simplify the model. Table S1 lists all the parameters that are kept fixed during the fitting. Beyond the parameters identified in the sensitivity analysis these are healthy cell parameters, which are assumed to be similar for all patients. The latter assumption is often used and usually does not compromise the agreement of model simulations and patient data[1,11].

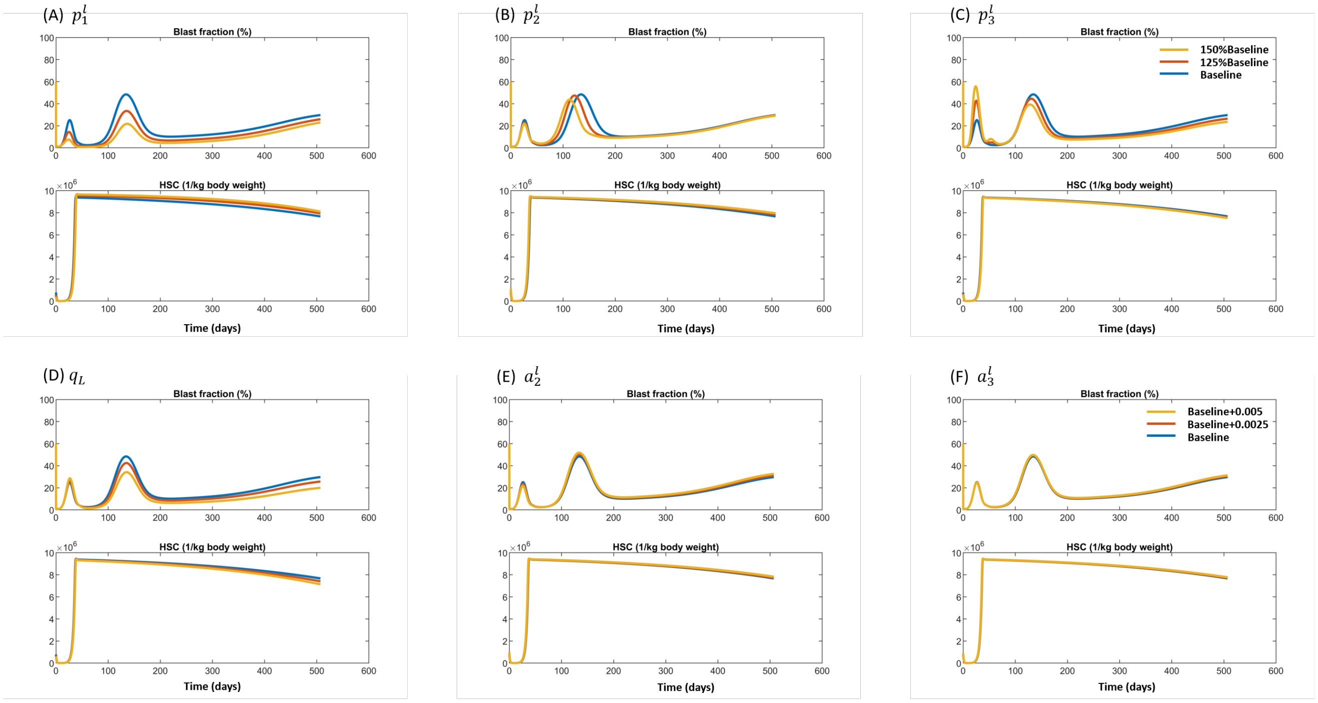


**Figure S2.** Figure (A) to (C) shows the sensitivity analysis with respect to perturbations of the leukemic cell proliferation rates ($p_1^l$,$p_2^l$,$p_3^l$); (A) LSC proliferation rate is changed from the baseline value which equals the equilibirum HSC proliferation to 125% and 150% of baseline value; (B) LProC proliferation rate is changed; (C) LPreC proliferation rate is changed. Figure (D) to (F) show the sensitivity analysis for HSC dislodgement probability ($q_L$) and self-renewal fraction of LProC and LPreC ($a_2^l$,$a_3^l$); (D) HSC dislodgement probability is changed from 0.99 to 0.9925 and 0.995; (E) LProC self-renewal fraction is changed; (F) LPreC self-renewal fraction is changed.

## S2.3 Patient data

We use data from a single study to ensure consistency of the HSC quantification. The clinical data taken from[2] consists of 4-7 longitudinal measurements of HSCs and blast fraction in bone marrow aspirates of 8 AML patients. We do not consider patients with less than 3 measurements. HSCs and blasts are quantified as percentages of the bone marrow mononuclear cells (MNCs). We exclude one patient from our study (Patient No.4) due to a simultaneous decrease of HSC and leukemic blast fraction in the post-treatment period. Such a dynamics cannot be explained by a competition between healthy and malignant cells and, therefore, cannot be reproduced by the proposed model.

## S2.4 Fitting

To facilitate the fitting of the model to data, we restrict the LSC proliferation rate $p_l^1$ and HSC dislodgment probability $q_L$ to plausible ranges. We need the LSCs' properties to satisfy the following two requirements:

**Requirement 1:** The time elapsing between the introduction of a small number of LSC to the healthy equilibrium and the presence of 20% marrow blasts is at most 10 years. 20% blasts are the key diagnostic criterion for AML. The 10 years are used as an upper bound of the pre-diagnostic disease phase[12, 13].

**Requirement 2:** If the marrow contains 80% blasts, the abundance of HSCs is still more than 1 cell/kg body weight. 80% marrow blasts are frequently observed in clinical routine[2]. Since patients with 80% marrow blasts can reach complete remission after 7+3 chemotherapy, their marrow has to harbor sufficient HSCs to restore healthy hematopoiesis. We numerically identified the ranges for $p_1^l$ and $q_L$ which satisfy these requirements. They are shown in Table S2.

To compare our model to clinical data, we have to calculate the blast frequency. Since post-mitotic leukemic blasts are assumed to be in the blood stream and LSCs are morphologically indistinguishable form healthy cells, we define the blast fraction $BF$ as the fraction of LProCs and LPreCs among all marrow cells:

$$BF = \frac{\sum_{i=0}^{n1} l_{2,i}(t) + \sum_{i=0}^{n2} l_{3,i}(t)}{\sum_{i=0}^{n1} l_{2,i}(t) + \sum_{i=0}^{n2} l_{3,i}(t) + l_1(t) + \sum_{i=0}^{n1} c_{2,i}(t) + \sum_{i=0}^{n2} c_{3,i}(t) + c_1(t)} \tag{S38}$$

| Parameters | Description | Value |
|---|---|---|
| $K$ | Niche capacity, i.e. maximum number of stem cells that can reside in the niche | $9.8\times10^{6}$ |
| $n$ | Maximum number of attempts stem cells undertake to occupy a niche space | 10 |
| $n_1$ | Maximum number of progenitor cells division before further differentiation | 30 |
| $n_2$ | Maximum number of precursor cells division before further differentiation | 20 |
| $p_1^c$ | Proliferation rate of healthy stem cells | 0.0152 |
| $p_2^c$ | Proliferation rate of healthy progenitor cells | 0.3961 |
| $p_3^c$ | Proliferation rate of healthy precursor cells | 0.6931 |
| $p_2^l$ | Proliferation rate of leukemic progenitor cells | 0.3961 |
| $p_3^l$ | Proliferation rate of leukemic precursor cells | 0.6931 |
| $a_2^c$ | Self-renewal fraction of healthy progenitor cells | 0.573 |
| $a_3^c$ | Self-renewal fraction of healthy precursor cells | 0.7399 |
| $a_2^l$ | Self-renewal fraction of leukemic progenitor cells | 0.573 |
| $a_3^l$ | Self-renewal fraction of leukemic precursor cells | 0.7399 |
| $d_4^c$ | Elimination rate of mature white blood cells | 0.01 |
| $k_p$ | Multiplicative parameter in feedback signal on proliferation rate | $9.677\times10^{-12}$ |
| $k_a$ | Multiplicative parameter in feedback signal on self-renewal fraction | $2.922\times10^{-13}$ |

**Table S1.** Model parameters kept fixed during the fitting. The parameters are taken from[1].

Since HSC counts in healthy individuals vary[2], we have to account for inter-individual differences in the niche size. The HSC frequency is defined as the percentage of HSCs among mononuclear marrow cells. We express the HSC frequency at time $t$ as $\widetilde{c}_{f1}(t) = (c_1(t)/K)K_{bm}$, where $c_1(t)$ is the number of HSCs per kg of body weight at time $t$, $K$ is the niche capacity and $K_{bm}$ is a calibration factor. The value of $K_{bm}$ should not be larger than the maximum of observed CD34+CD38-ALDH+ cell frequencies in clincial data which is 0.6% of the total mono-nuclear cells[2]. Since the individual niche capacities are not known and the HSC counts vary considerably in healthy individuals[2], we fit the parameter $K_{bm}$ based on the individual data. Table S1 shows all parameters which are fixed during the fitting. Table S2 lists all the parameters that are varied to fit the model to individual patients' data. We used the nonlinear optimization function "fmincon" from MATLAB to minimize the value of a cost function, which in our case is the sum of squared differences between simulation results and clinical data, see equation S39:

$$sum\ of\ squared\ residuals = \sum(\widetilde{c}_1(t_i) - c_1(t_i))^2 + \sum(\widetilde{BF}(t_i) - BF(t_i))^2 \tag{S39}$$

For each of the patients, we use approximately hundred initial guesses to start the optimization and iteratively use the obtained optimization result as the initial guess again until the objective values are identical[14]. The obtained parameters are listed in Table S3 To double-check the optimization procedure we used a bruit force approach. We generated 1000 random parameter sets which fulfill the imposed constraints using Latin-Hypercube sampling. Parameter sets leading to small values of the cost function are compared to the previously found optima to check if they lead to a better approximation of the data. Figure S3 illustrates the optimal trajectory for patient No.8 in comparison to trajectories obtained for randomly sampled parameter values.

## S3 Model dynamics without the niche-related feedback signal

To test how quickly HSC can regenerate in absence of the niche-related feedback, we simulate an idealized therapy which immediately removes all leukemic cells at the time of diagnosis and has no side effects on hematopoietic cells. Figure S4 illustrates the difference between the time evolution of cells with and without the niche-related feedback signal ($p_l^1/\tilde{p}_c^1 = 12, q_L = 0.92, BF = 80\%$). In Fig.S4(A), we observe that HSCs take more than three years to recover and the mature cells exhibit an unrealistic delayed decline. Even when increasing the response of the HSCs to the systemic signal by a factor of 5, a late temporary decline of mature cells is observed, Fig.S4 (B). When accounting for the niche-related feedback signal (see Fig. S4(C)), HSCs recover faster to the equilibrium state and cause no decline of progenitor, precursor and mature blood cells. From

| Parameter | Description | Possible range |
|---|---|---|
| $p_1^l$ | Proliferation rate of LSCs. | no faster than $12\widetilde{p_1^c}\star$ |
| $q_L$ | HSC dislodgement probability, i.e. probability that LSC dislodges HSC from the niche. | $(0.9, 1)$ |
| $k_{arac}$ | Multiplicative constant relating a cell type's proliferation rate to its AraC induced elimination. If the proliferation rate of a cell type is $p$, the drug AraC induced death rate is $k_{arac}p$. | depends on $p_1^l$ |
| $k_{dnr}$ | Drug DNR induced cell death rate. | $> 0$ |
| $s_{max}$ | Maximum value of the stimulation signal regulated by the empty niche space. | $> 0$ |
| $d_4^l$ | Elimination rate of leukemic post-mitotic blasts. | $> 0$ |
| $K_{bm}$ | Calibration value for individual patient's niche capacity. | $(0.1, 0.6)$ |

**Table S2.** Model parameters which are varied when the model is fitted to patient data. $\star\widetilde{p_1^c}$ denotes the proliferation rate of HSC in healthy equilibrium state.

| Patient No. | $q_L$ | $p_1^l/\widetilde{p^c}_1$ | $k_{arac}$ | $k_{dnr}$ | $s_{max}$ | $d_4^l$ | $K_{bm}$ |
|---|---|---|---|---|---|---|---|
| 3 | 0.99172 | 1.0386 | 299.15 | 0.11856 | 38.3722 | 0.01 | 0.35 |
| 5 | 0.993 | 1.5 | 290 | 0.09 | 14.5 | 0.44 | 0.22 |
| 6 | 0.99489 | 1.8775 | 144.4531<br>75$\star$ | 0.62049 | 18.4319 | 0.40356 | 0.09 |
| 7 | 0.99999 | 1.8443 | 152.2515 | 0.34625 | 30.2965 | 0.01 | 0.6 |
| 8 | 0.99999 | 1.0507 | 115.3172 | 0.01 | 34.0295 | 0.052783 | 0.12 |
| 9 | 0.99979 | 1.1572 | 129.5677 | 0.074985 | 25.0986 | 0.24171 | 0.11229 |
| 10 | 0.99999 | 0.71793 | 124.6324 | 0.053273 | 29.3688 | 0.012074 | 0.10101 |

**Table S3.** Parameter estimation results for each patient. $\widetilde{p^c}_1$ refers to the proliferation rate of HSC in healthy equilibrium state. As LSCs proliferate faster than HSCs[15], we list the ratio between LSC and HSC proliferation rates. $\star$ For patient 6 we simulate a salvage scheme since blast fractions increase within the first 4 weeks after initiation of therapy. We simulate 3 days of AraC starting at the 42nd day after diagnosis.

our test, as long as parameter $s_{max}$ is larger than 5, there will be no significant decline in the time evolution of HProCs, HPreCs and mature blood cells. In summary, adding our niche-related feedback signal can overcome the unrealistic decline of mature blood cells.

### S3.1 Mature blood cells and post-mitotic blasts simulation results

Figure S5 illustrates the time evolution of leukemic post-mitotic blasts (blue lines) and healthy mature blood cells (red lines) for the simulations shown in Fig.2. Mature blood cells abundance recovers to a healthy equilibrium state level around 100 days after chemotherapy, which is in the time range observed in clinical data[16].

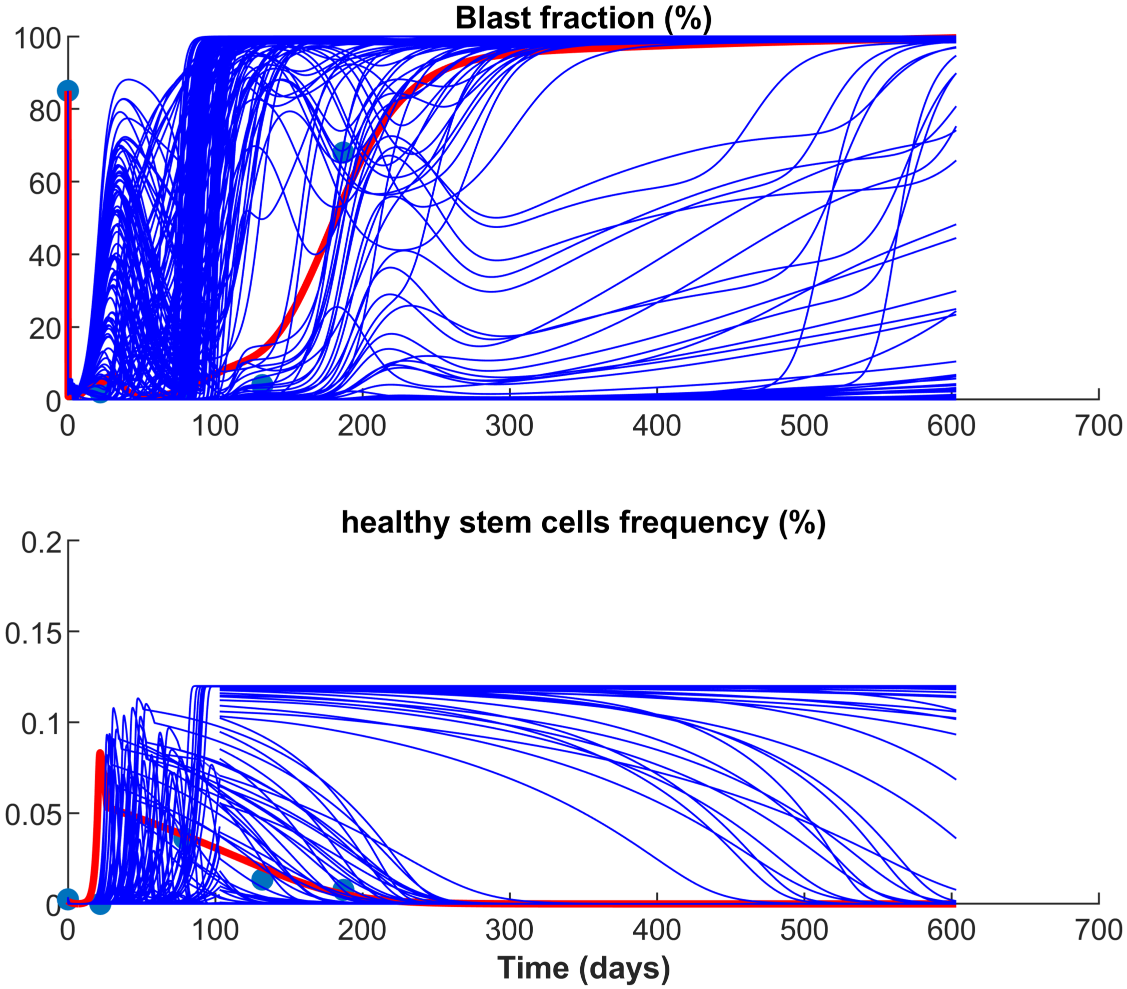


**Figure S3.** Model simulations with thousand random parameter sets generated by Latin-Hypercube sampling (blue lines). Blue dots are the clinical data of patient No.8 in[2] and the red line is the final fitting results from Fig.2(E).

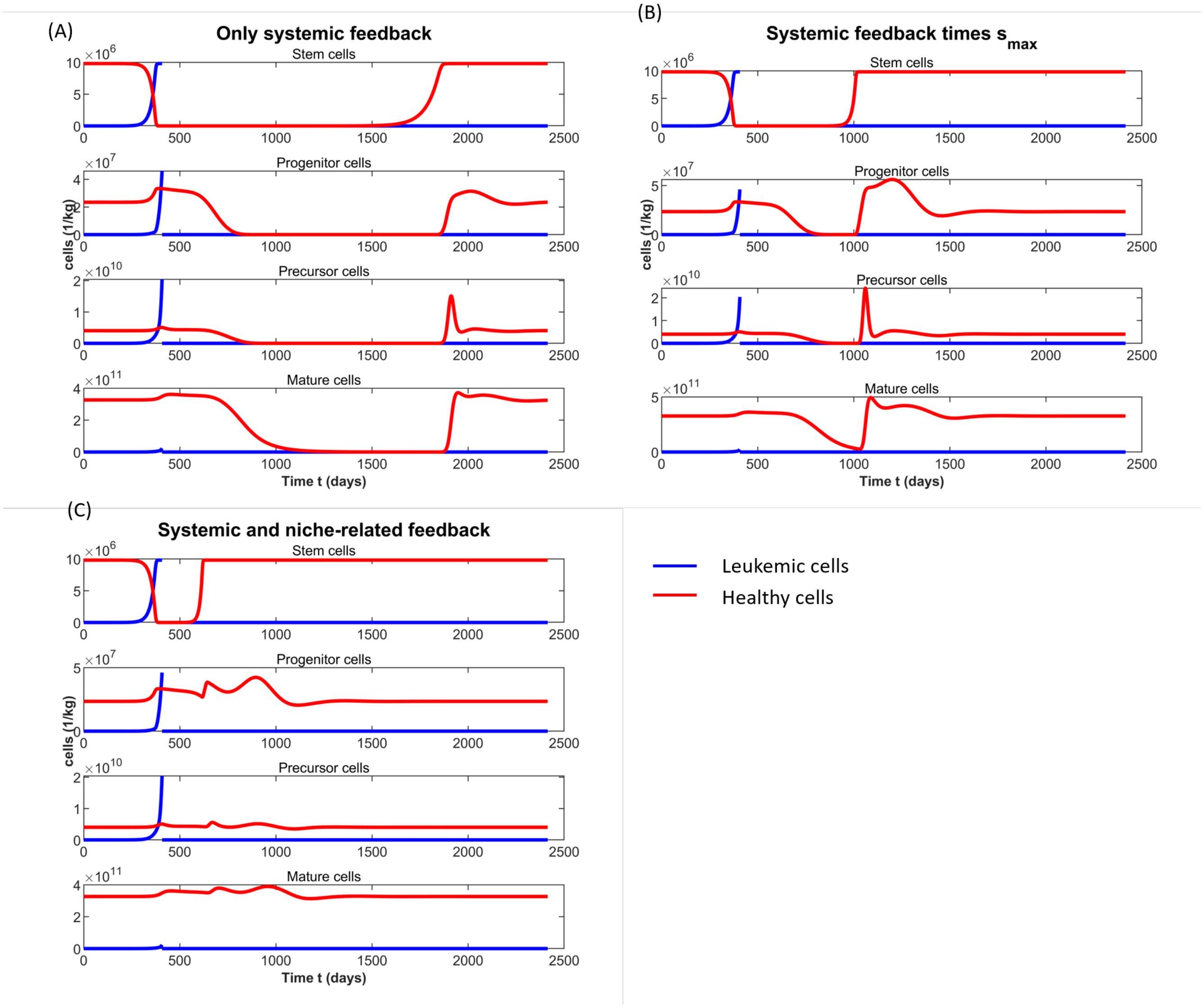


**Figure S4.** Comparison of the impact of systemic and niche-related feedback. We consider an idealized therapy. We assume that the disease is diagnosed when the blast counts exceed 80%. At this timepoint we immediately eliminate all leukemic cells and let healthy hematopoiesis recover. Parameters: $p_l^1/\tilde{p}_c^1 = 12$, $(q_L = 0.92)$. (A) shows the time evolution of cells if only the systemic feedback is present. (B) shows the time evolution of cells when we amplify the effect of the systemic signal by the factor of $s_{max}$, i.e., 5, but still do not consider the niche-related feedback. (C) shows the time evolution of cells in presence of the systemic and the niche-related feedback.

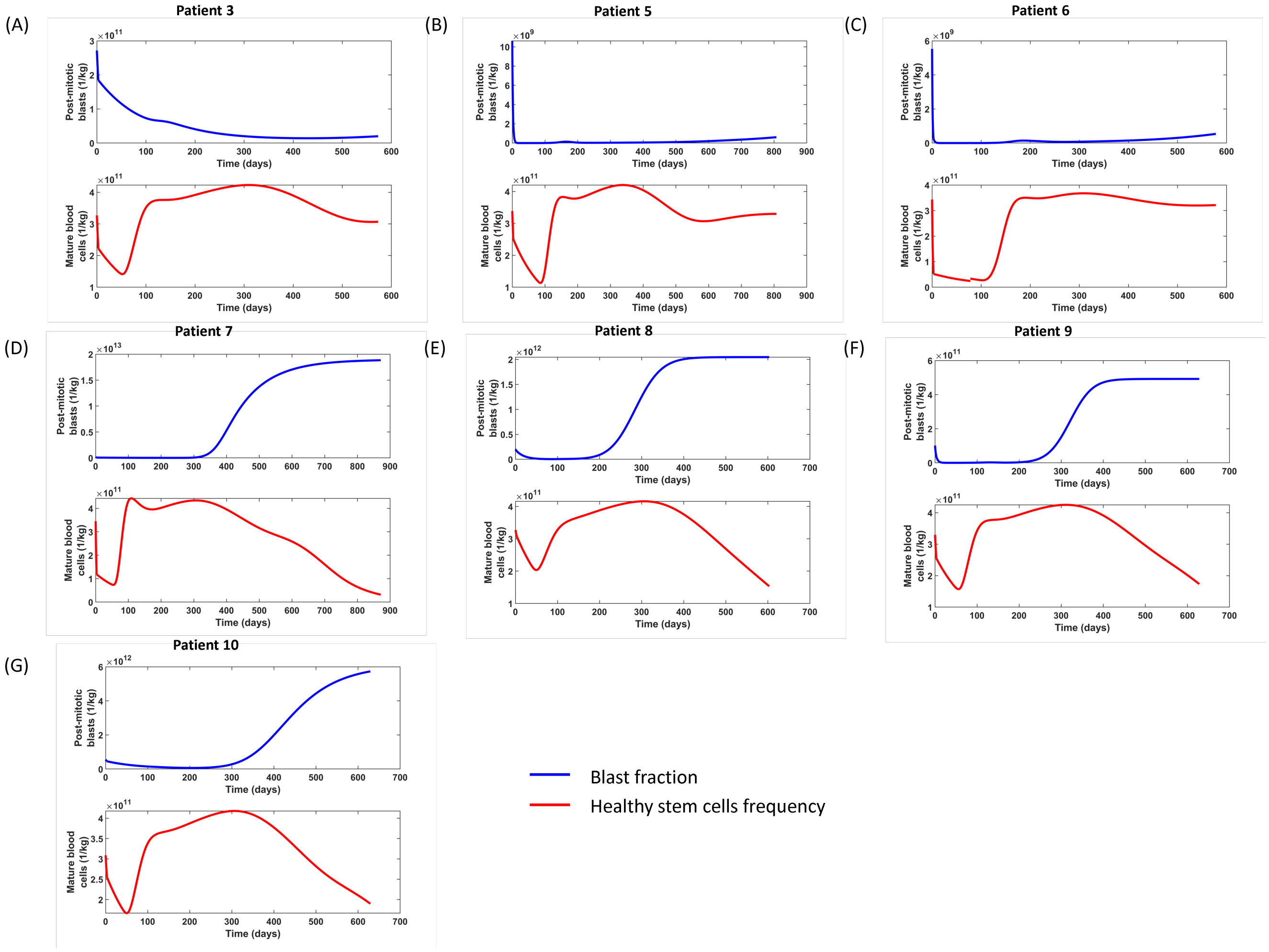


**Figure S5.** Simulation results of the time evolution of post-mitotic blasts and mature blood cells dynamics for the scenarios considered in Fig.2. In each of the panel, the upper graph with blue line illustrates the time evolution of leukemic post-mitotic blasts and the below graph with red line shows that of mature blood cells.

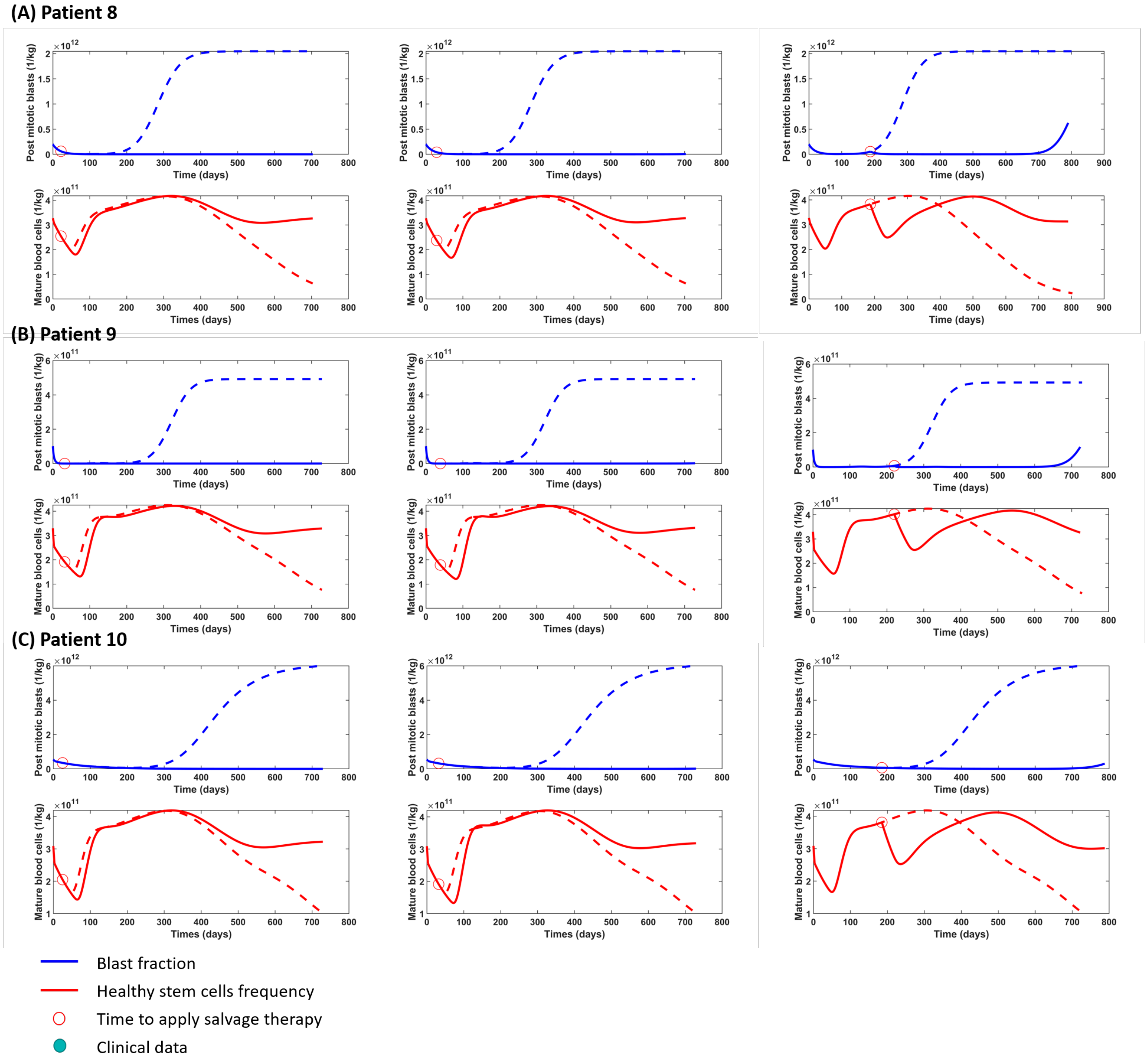


**Figure S6.** Time evolution of post-mitotic cells during salvage therapy. Comparison of different indications for salvage therapy. We consider the patients from Fig.2 (E)-(G). The dashed lines indicate the disease evolution in absence of salvage therapy. They are identical to the simulations in Fig.2 (E)-(G). In the left column therapy is initiated at the time, when the HSC counts start to decline. In the second column therapy is initiated one week after HSC counts started to decline. In the third column salvage therapy is initiated at the time of the first clinical measurement reporting more than 5% blasts. Red circles mark the time point where salvage therapy starts. Simulation results in Panel (A) using same parameters set and clinical data as in Fig.2 (E), Panel (B) corresponds to Fig.2 (F), and Panel (C) is corresponds to Fig.2 (G). Immature cell dynamics are shown in Fig.6.

## S4 Extended Models

### S4.1 Simulation of HSC survival after dislodgement

In the original model, HSCs will start to differentiate when they have been dislodged from the bone marrow niche. However, recent studies from[17,18] emphasize the contribution of circulating HSCs to human hematopoiesis. To model the prolonged survival of HSCs after dislodgement, we use the AML model without chemotherapy ($d_i^{chemo} = 0$) and let 25% of dislodged HSCs return to the stem cell compartment to compete again for niche spaces. The modified equations are provided below (equations (S40) and (S41)), $v_1^c$ is the flux of cells from HSC to HProC from equation (S5). Simulation results are shown in Fig. S7, where (A) and (B) are the results for the scenario where 25% of the dislodged cells return to the stem cell compartment and (C) and (D) are the respective results for the original model.

To identify the healthy equilibrium state we start the simulations with the HSC number equal to the niche capacity and other cells equal to zero, i.e., $c_1(t=0) = K, c_{2,3,4}(t=0) = 0$,and $l_{1,2,3,4}(t=0) = 0$, and let the system converge to the physiological equilibrium, (see Fig. S7 (A) and (C)). When the system has reached the equilibrium state up to numerical precision, we add one LSC per kg of body weight to the system and let the disease evolve (see Fig. S7 (B) and (D)). We observe that the dynamics of both model versions are very similar.

$$\begin{aligned}\frac{d}{dt}c_1 = &\left(1-\left(1-\frac{K-c_1-l_1}{K}-q_H\frac{l_1}{K}\right)^n\right)p_1^c c_1 \\ &-\left(1-\left(1-\frac{K-c_1-l_1}{K}-q_L\frac{c_1}{K}\right)^n\right)\frac{c_1 q_L}{K-l_1-c_1+q_L c_1}p_1^l l_1 + 0.25v_1^c\end{aligned} \quad \text{(S40)}$$

$$\frac{d}{dt}c_{2,0} = 0.75v_1^c - p_2^c c_{2,0} \quad \text{(S41)}$$

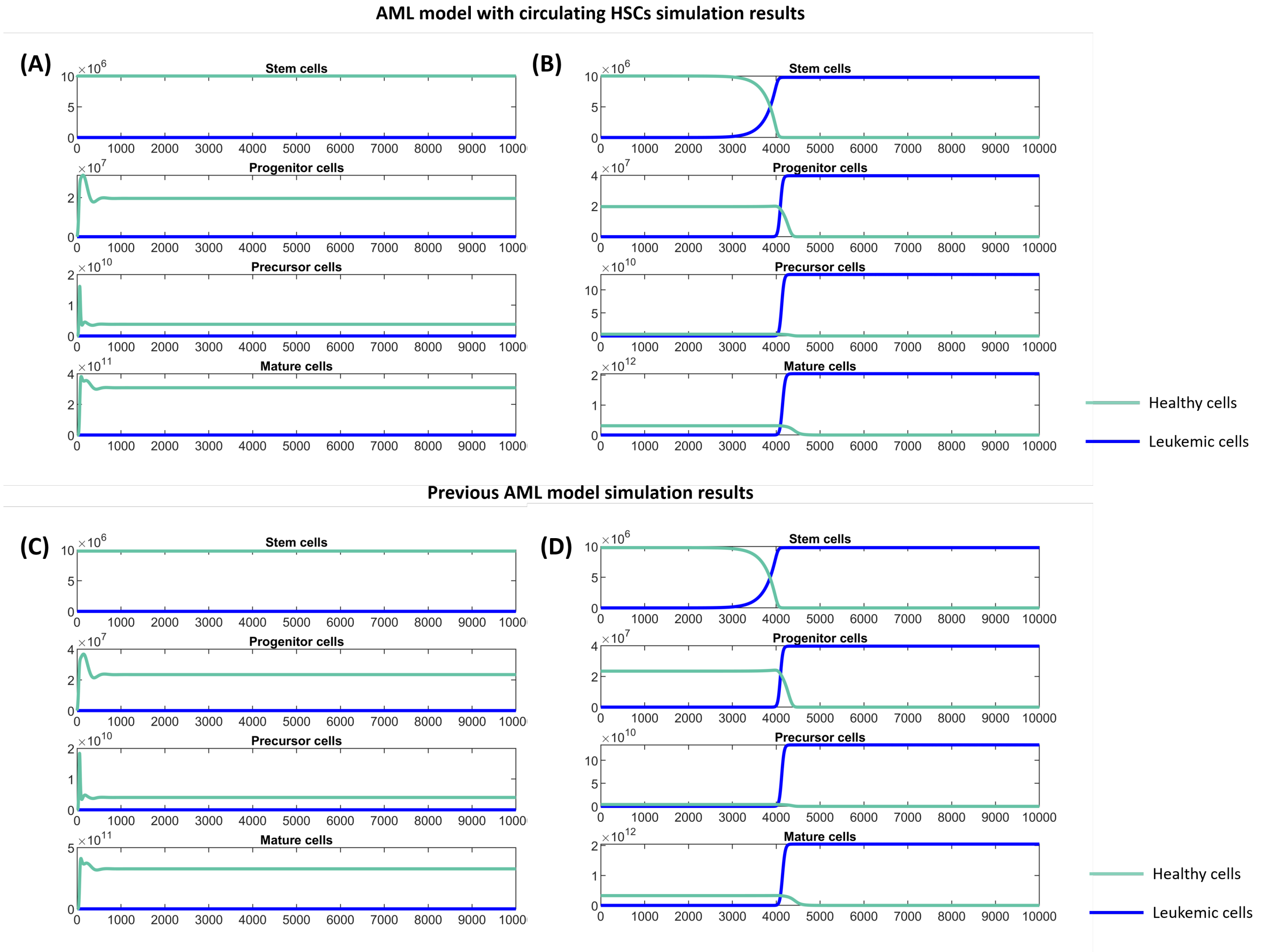


**Figure S7.** Comparison of the simulation results from the original model and the model with prolonged HSC survival after dislodgement. (A) and (B) are the simulation results accounting for prolonged survival of HSCs after dislodgement, where (A) shows the convergence to the healthy equilibrium in absence of leukemic cells and (B) shows disease evolution after one LSC per kg of body weight has been added to the healthy equilibrium. (C) and (D) depict the simulation results of the original model, (C) shows the convergence to the healthy equilibrium and (D) the disease progression.

## S4.2 Impact of AML blasts on healthy blood cell formation

### *Paracrine IL6 signaling of AML blasts induces cytopenia*

Recent studies from[19] demonstrate that AML blasts suppress healthy blood cell formation by paracrine signaling via IL6. The paracrine signals lead to a reduction of erythroid progenitors and precursors. We extended our model to perform proof of principle simulations for this mechanism. For this purpose, we modify the feedback signal which regulates the cell fluxes during the maturation of progenitors and precursors, i.e., the higher the concentration of the paracrine signals, the lower the numbers of cells entering committed cell compartments. In our model the cellular flux of progenitors and precursors during maturation is regulated by a feedback signal $s_a(t) = \frac{1}{1+k_a c_4(t)}$, see Section S1.3 of the Supplement and equation (S19). To model the impact of paracrine signaling by the AML blast population $l_4(t)$, we replace the signal $s_a(t)$ by

$$\tilde{s}_a(t) = \frac{1}{1 + k_a c_4(t) + k_{IL6} l_4(t).} \tag{S42}$$

The positive constant $k_{IL6}$ is quantifies the secretion of paracrine factors. The higher the secretion, the higher the suppression of healthy progenitors and precursors. In agreement with experimental observations[19], we assume that the paracrine signaling does not affect apoptosis or proliferation rate. This modification implies that the presence of AML blasts reduces the numbers of progenitor and precursor cells, as experimentally observed for the red lineage. Simulation results for the models with and without paracrine signaling are illustrated in Fig. S8. We observe that the IL6 secretion by AML blasts induces peripheral cytopenia around 300 days after chemotherapy (solid red line in panel (B)).

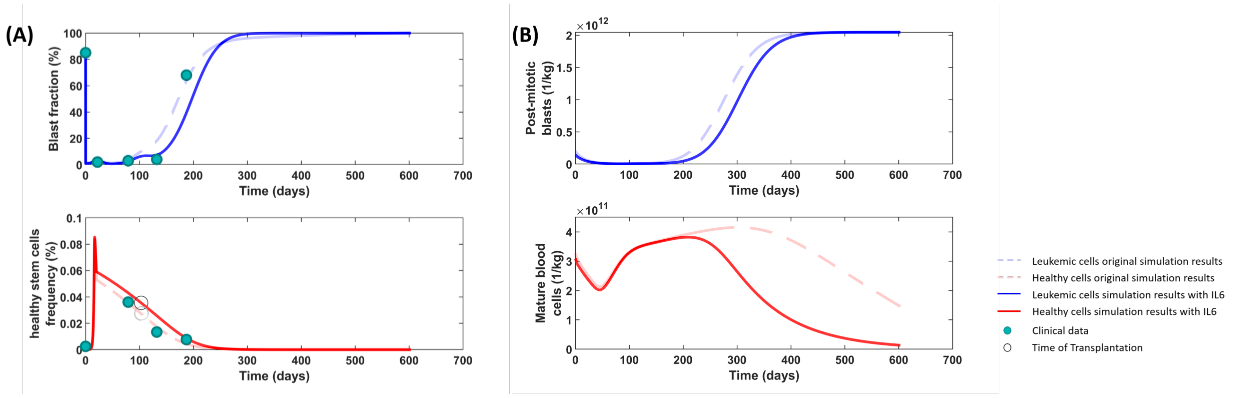


**Figure S8.** Simulation of the model accounting for the suppression of healthy hematopoiesis by paracrine factors from AML blasts. (A) illustrates the time evolution of the blast fraction and healthy stem cells frequency. (B) illustrates the time evolution of leukemic post-mitotic blasts and mature blood cells. Both dashed lines correspond to the original simulation results from Fig.2 (E) and the solid lines show simulation results of the modified model with the signal $\tilde{s}_a$.

### *Perturbation of cytokine feedback by MPL expression on AML blasts*

There is evidence that the expression of the cytokine receptor MPL on AML blasts reduces the thrombopoietin (TPO) concentration and thus aggravates peripheral neutropenia and thrombocytopenia[20]. This mechanism can explain why patients with high MPL expression on AML blasts have less peripheral thrombocytes and neutrophils compared to patients with low MPL expression on AML blasts. We extend our model to check whether it can qualitatively reproduce these observations. In our model, we use equation (S12) to simulate the impact of the systemic signals on cell proliferation and equation (S19) to simulate the impact of systemic signals on progenitor and precursor maturation. To account for the reduction of the systemic feedback signal concentrations by MPL expressed on AML blasts we replace $s_1(t)$ (equation (S12)) by

$$\tilde{s}_1(t) = \frac{1}{1 + k_p c_4(t) + k_{mpl} l_4(t)} \tag{S43}$$

and $s_a(t)$ (equation (S19)) by

$$\tilde{s}_a(t) = \frac{1}{1 + k_a c_4(t) + k_{mpl} l_4(t)}. \tag{S44}$$

Here, $k_{mpl}$ is a positive constant, which describes the elimination of the systemic signal by receptors on AML blasts. The higher the expression of MPL on AML blasts, the higher the value of $k_{mpl}$. We observe that high blast counts and high MPL

expression result in low concentrations of the feedback signal.

Simulation results are illustrated in Fig. S9. It compares the simulated disease progression of the models with and without MPL expession on AML blasts. We observe that the expression of MPL receptors on AML blasts triggers peripheral cytopenia around 300 days after chemotherapy (solid red line in panel (B)).

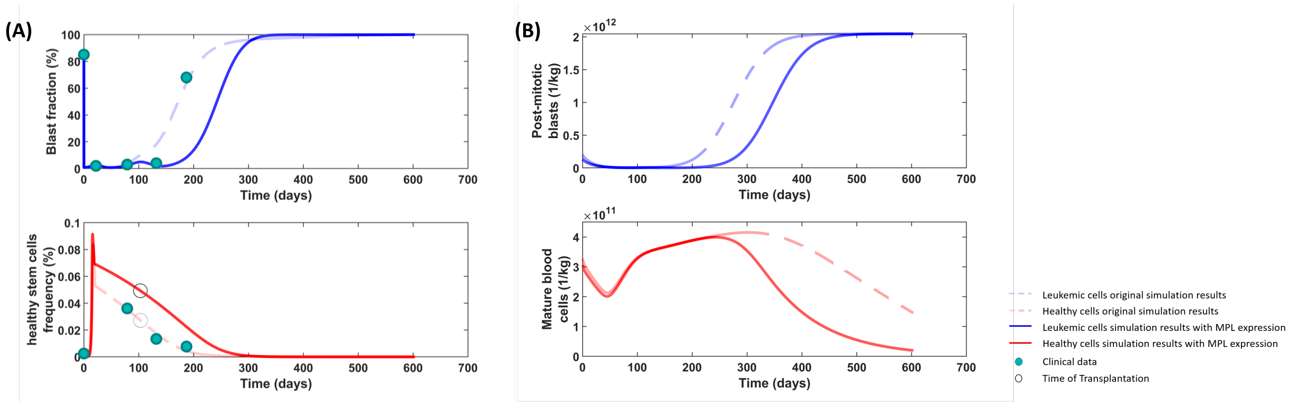


**Figure S9.** Simulation of the model accounting for the expression of cytokine receptors on AML blasts. (A) illustrates the time evolution of the blast fraction and healthy stem cell frequency. (B) illustrates the time evolution of leukemic post-mitotic blasts and mature blood cells. Both dashed lines correspond to the original simulation results from Fig.2 (E) and the solid lines show simulation results of the extended model accounting for the perturbation of systemic feedback signals by AML blasts.

## References


1. Stiehl, T., Wang, W., Lutz, C. & Marciniak-Czochra, A. Mathematical modeling provides evidence for niche competition in human aml and serves as a tool to improve risk stratification. *Cancer Res* **80**, 3983–3992, DOI: 10.1158/0008-5472.CAN-20-0283 (2020).
2. Wang, W. *et al.* Reduced hematopoietic stem cell frequency predicts outcome in acute myeloid leukemia. *Haematologica* **102**, 1567–1577, DOI: 10.3324/haematol.2016.163584 (2017).
3. Boyd, A. L. *et al.* Niche displacement of human leukemic stem cells uniquely allows their competitive replacement with healthy hspcs. *J Exp Med* **211**, 1925–35, DOI: 10.1084/jem.20140131 (2014).
4. Kaushansky, K. *Hematopoietic Stem Cells, Progenitors, and Cytokines*, chap. 18 (McGraw-Hill Education, New York, NY, 2021).
5. Hayakawa, F. *et al.* Tandem-duplicated Flt3 constitutively activates STAT5 and MAP kinase and introduces autonomous cell growth in IL-3-dependent cell lines. *Oncogene* **19**, 624–631, DOI: 10.1038/sj.onc.1203354 (2000).
6. Liu, R. Y., Fan, C., Garcia, R., Jove, R. & Zuckerman, K. S. Constitutive activation of the JAK2/STAT5 signal transduction pathway correlates with growth factor independence of megakaryocytic leukemic cell lines. *Blood* **93**, 2369–2379 (1999).
7. Komarova, N. L., Sadovsky, A. V. & Wan, F. Y. M. Selective pressures for and against genetic instability in cancer: a time-dependent problem. *J. Royal Soc. Interface* **5**, 105–121, DOI: 10.1098/rsif.2007.1054 (2008).
8. Stiehl, T., Baran, N., Ho, A. D. & Marciniak-Czochra, A. Clonal selection and therapy resistance in acute leukaemias: mathematical modelling explains different proliferation patterns at diagnosis and relapse. *J R Soc Interface* **11**, 20140079, DOI: 10.1098/rsif.2014.0079 (2014).
9. Hamby, D. M. A review of techniques for parameter sensitivity analysis of environmental models. *Environ Monit Assess* **32**, 135–54, DOI: 10.1007/BF00547132 (1994).
10. Stiehl, T. Using mathematical models to improve risk-scoring in acute myeloid leukemia. *Chaos* **30**, 123150, DOI: 10.1063/5.0023830 (2020).
11. Stiehl, T., Baran, N., Ho, A. D. & Marciniak-Czochra, A. Cell division patterns in acute myeloid leukemia stem-like cells determine clinical course: a model to predict patient survival. *Cancer Res.* **75**, 940–949, DOI: 10.1158/0008-5472.CAN-14-2508 (2015).

**12.** Abelson, S. *et al.* Prediction of acute myeloid leukaemia risk in healthy individuals. *Nature* **559**, 400–404, DOI: 10.1038/s41586-018-0317-6 (2018). Number: 7714, Publisher: Nature Publishing Group.

**13.** Desai, P. *et al.* Somatic mutations precede acute myeloid leukemia years before diagnosis. *Nat. Medicine* **24**, 1015–1023, DOI: 10.1038/s41591-018-0081-z (2018). Number: 7, Publisher: Nature Publishing Group.

**14.** Kreutz, C. Guidelines for benchmarking of optimization-based approaches for fitting mathematical models. *Genome Biol* **20**, 281, DOI: 10.1186/s13059-019-1887-9 (2019).

**15.** Grove, C. S. & Vassiliou, G. S. Acute myeloid leukaemia: A paradigm for the clonal evolution of cancer? *Dis. Model. & Mech.* **7**, 941–951, DOI: 10.1242/dmm.015974 (2014).

**16.** Jost, F., Schalk, E., Rinke, K., Fischer, T. & Sager, S. Mathematical models for cytarabine-derived myelosuppression in acute myeloid leukaemia. *PLOS ONE* **14**, e0204540, DOI: 10.1371/journal.pone.0204540 (2019). Publisher: Public Library of Science.

**17.** Quaranta, P. *et al.* Circulating hematopoietic stem/progenitor cell subsets contribute to human hematopoietic homeostasis. *Blood* **143**, 1937–1952, DOI: 10.1182/blood.2023022666 (2024).

**18.** Mende, N. *et al.* Unique molecular and functional features of extramedullary hematopoietic stem and progenitor cell reservoirs in humans. *Blood* **139**, 3387–3401, DOI: 10.1182/blood.2021013450 (2022).

**19.** Zhang, T. Y. *et al.* IL-6 blockade reverses bone marrow failure induced by human acute myeloid leukemia. *Sci. Transl. Medicine* **12**, eaax5104, DOI: 10.1126/scitranslmed.aax5104 (2020).

**20.** Rauch, P. J. *et al.* MPL expression on AML blasts predicts peripheral blood neutropenia and thrombocytopenia. *Blood* **128**, 2253–2257, DOI: 10.1182/blood-2016-04-711986 (2016).
